# Swarm coordination of fish-like magnetic soft robots: directed aggregation and shape-adaptive attachment toward efficient drug delivery

**DOI:** 10.1093/nsr/nwaf429

**Published:** 2025-10-21

**Authors:** Liyang Mao, Chenyao Tian, Peng Yang, Xianghe Meng, Xingjian Shen, Hao Zhang, Hui Xie

**Affiliations:** State Key Laboratory of Robotics and Systems, School of Mechatronics Engineering, Harbin Institute of Technology, Harbin 150001, China; State Key Laboratory of Robotics and Systems, School of Mechatronics Engineering, Harbin Institute of Technology, Harbin 150001, China; State Key Laboratory of Robotics and Systems, School of Mechatronics Engineering, Harbin Institute of Technology, Harbin 150001, China; State Key Laboratory of Robotics and Systems, School of Mechatronics Engineering, Harbin Institute of Technology, Harbin 150001, China; State Key Laboratory of Robotics and Systems, School of Mechatronics Engineering, Harbin Institute of Technology, Harbin 150001, China; State Key Laboratory of Robotics and Systems, School of Mechatronics Engineering, Harbin Institute of Technology, Harbin 150001, China; State Key Laboratory of Robotics and Systems, School of Mechatronics Engineering, Harbin Institute of Technology, Harbin 150001, China

**Keywords:** magnetic untethered soft robots, fish-like locomotion, swarm coordination, directed aggregation, shape-adaptive attachment

## Abstract

Miniature magnetic untethered soft robots offer promising opportunities for biomedical applications due to their tissue compatibility, functionalizable dimensions and flexible locomotion capabilities. However, their small size creates a mismatch between coverage area and lesion regions, limiting drug delivery efficacy. Here, inspired by natural fish migration and foraging behaviors, we present an approach to overcome these limitations through swarm coordination of fish-like magnetic soft robots. Individual robots with simplified designs for scalable production can perform constrained six-degrees-of-freedom fish-like maneuvers under an oscillating magnetic field and gradient magnetic field. By exploiting the unique property of the constant component of the oscillating magnetic field dominating the swimming direction when the actuation frequency approaches the robot’s natural frequency, we achieved differentiated control of individual swimming directions under global magnetic field actuation through programming spatial distribution patterns of the constant field component. This enables our robotic swarm to demonstrate active spatial aggregation toward specific lesion areas and shape-adaptive attachment capabilities upon reaching the target. This work provides a new pathway for efficient therapeutic payload delivery to lesions through coordinated miniature magnetic soft robot swarms.

## Teaser

Programmed magnetic fields with specific oscillation frequencies coordinate miniature swimming robots into 3D fish-like schools, offering potential for targeted therapeutic delivery in clinical settings.

## INTRODUCTION

Miniature magnetic untethered soft robots, with their tissue-compatible elastic moduli, functionalizable modest dimensions and flexible locomotion capabilities, can navigate smoothly through complex lumina, offering possibilities for clinical applications such as drug delivery [1–4]. However, while the small size of individual robots enhances accessibility, it also creates a mismatch between effective coverage area and lesion regions, limiting the adequacy of drug delivery dosages. Fortunately, collective systems formed by artificial individuals through local interactions and environmental responsiveness can emerge with efficiency, adaptability and robustness beyond individual capabilities, providing new approaches to complex challenges that single robots cannot address [5,6].

However, swarm coordination methods suitable for miniature magnetic untethered soft robots remain significantly inadequate, limiting their ability to form swarms that effectively cover lesions and deliver sufficient medication. On the one hand, size-constrained miniature robots cannot integrate autonomous hardware and primarily rely on external magnetic field propulsion. The globally consistent magnetic field signals in the workspace result in identical control inputs across all robots [7,8], hindering effective coordination and impeding functional swarm formation (Fig. S1a). Although individual response differences [9,10] or magnetic field spatial distribution [11,12] can achieve independent control of a few robots, extending such control to the swarm level still faces major challenges (Fig. S1b). On the other hand, magnetic untethered soft robots operating in transitional Reynolds number environments involve complex movement mechanisms, rendering coordination strategies for simple micro/nanoparticle swarms that depend on equilibrated physical field interactions in low Reynolds number environments no longer applicable (Fig. S1c) [13–18].

**Figure 1.**
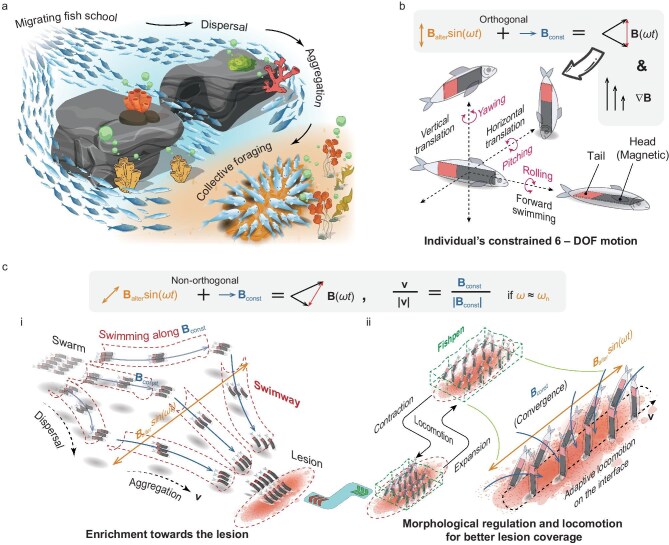
The miniature fish-like magnetic soft robot swarm: its aggregation and optimal coverage of lesions. (a) In nature, small fish can swim agilely and increase their survival probabilities by forming schools that dynamically aggregate and disperse for collective foraging. (b) Inspired by nature, a miniature fish-like magnetic soft robot composed of hard-magnetic elastomers has been developed. Under the actuation of an oscillating magnetic field **B**(*ωt*)—synthesized from perpendicular constant magnetic component **B**_const_ and alternating magnetic component **B**_alter_sin(*ωt*)—and with the assistance of a gradient magnetic field ▽**B**, the robotic individuals can achieve fish-like swimming with constrained 6 DOF, including pitch, yaw, roll, and horizontal and vertical translation, as well as forward swimming. (c) Based on the mechanism where the constant component **B**_const_ of an oscillating magnetic field at natural frequency *ω*_n_ uniquely dominates the swimming direction of fish-like magnetic soft robots, there is no need to satisfy orthogonality requirements between constant and alternating components. By programming the spatial distribution pattern of the constant component **B**_const_, the swimming direction of individual robots can be differentially regulated, inducing swarm behaviors. (i) Robotic swarms can disperse three-dimensionally and aggregate along swimways (migration pathways utilized by fish schools) like migrating fish schools, achieve obstacle avoidance, and enrich toward the lesion. (ii) Upon reaching the lesion interface, the robot swarm can then adjust its morphology and position on the solid–liquid interface, similar to fish foraging in a fishpen (confined feeding areas where fish gather), adapting to the lesion boundary for better adhesion.

Inspired by fish migration and foraging behaviors (Fig. 1a), we present miniature fish-like magnetic soft robots and their swarm coordination method, achieving directional aggregation toward target lesions and shape-adaptive attachment functionality. Individual robots are made from hard magnetic elastomer materials with simplified designs for scalable production. Under an oscillating magnetic field **B**(*ωt*) synthesized from orthogonal constant field **B**_const_ and alternating field **B**_alter_sin(*ωt*), supplemented by gradient field ∇**B** control, the robotic individual can perform agile maneuvers—such as pitching, yawing, rolling and high-speed forward swimming—similar to fish using tail propulsion, as well as horizontal and vertical translations akin to fish using pectoral propulsion, cumulatively offering constrained six degrees of freedom (DOF) (lacking only backward swimming) (Fig. 1b). Based on the experimentally discovered feature whereby the constant component **B**_const_ of the oscillating magnetic field uniquely dominates the swimming direction of fish-like magnetic soft robots when the actuation field frequency (*ω*) approaches the robot’s natural frequency (*ω*_n_), we achieve differentiated control of individual swimming directions under global magnetic field actuation by programming spatial distribution patterns of the constant field component. This enables the formed fish-like magnetic soft robots to swarm, demonstrating active spatial aggregation and dispersal capabilities similar to migrating fish schools in a swimway, allowing targeted gathering toward specific lesion areas (Fig. 1c i). Upon reaching the lesion plane, the robot swarm can actively adjust its morphology and position like foraging fish schools in a fishpen, adapting to the lesion boundary shape for effective attachment (Fig. 1c ii). Compared to existing robot swarm (Table 1), this system demonstrates faster swimming speeds and enhanced maneuverability at the individual level (Fig. S2), while at the collective level it combines the spatial movement capabilities of macroscopic robot groups with the scale advantages of microscopic particle robot collectives, providing a new technological pathway for targeted drug delivery to lesions.

**Table 1.** Comparison of our work with existing robots and swarms at various scales.

		Individual performance				Swarm performance				
Unit size	Reference	Actuation strategy	Motion style	Speed^a^ (BL/s)	Mobility	Swarm strategy	Swarm formation	Quantity	Domain	Collective behaviors
Micro scale	Xie *et al.* [13]	Magnetic	Rolling	∼8	2 DOF (2D)	Self-organization under stimuli	Yes	Collective level	Underwater (2D)	Morphological and positional regulation
	Magdanz *et al.* [19] Middelhoek *et al.* [20]	Magnetic	Ciliary propulsion	<1	2 DOF (2D)	Self-organization into clusters	Yes	Collective level	Underwater (2D)	Rotate and roll as a whole
	Yan *et al.* [14]	Electric	Self-propulsion		1 DOF (2D)	Self-organization under stimuli	Yes	Collective level	Underwater (2D)	Morphological regulation
	Palacci *et al.* [15]	Optical	Self-propulsion		1 DOF (2D)	Self-organization under stimuli	Yes	Collective level	Underwater (2D)	Morphological regulation
	Loghin *et al.* [16] Gwisai *et al.* [17]	Biodynamic^b^ (uncontrollable)	Cruise	∼10	3 DOF (2D)	Converging magnetic fields	Yes	Collective level	Underwater (3D)	Aggregation
	Wu *et al.* [8]	Magnetic	Corkscrew	∼7	3 DOF (3D)		No	Collective level	Underwater (3D)	Unable to maintain swarm stability
Millimeter scale	Hu *et al.* [1]	Magnetic	Undulating	**∼20**	3 DOF (3D)		No			
	Ren *et al.* [3]	Magnetic	Jellyfish-like	10	3 DOF (3D)		No			
	Wang *et al.* [2]	Magnetic	Swimming	∼15	3 DOF (3D)		No			
	Huang *et al.* [21]	Magnetic	Helical propulsion	∼4	3 DOF (3D)		No			
	Chaluvadi *et al.* [22]	Magnetic	Helical propulsion	<1	3 DOF (3D)	Converging magnetic fields	Yes	9	In agar gel (3D)^d^	Dispersal, aggregation
	Xu *et al.* [23]	Magnetic	Gradient force	<1	6 DOF (3D)		No			
	Xu *et al.* [9]	Magnetic	Stick-slip	∼2	3 DOF (3D)	Independent control	No	4		
	Diller *et al.* [10]	Magnetic	Gradient force	<1	5 DOF (3D)	Independent control	No	2		
	Ongaro *et al.* [12]	Magnetic	Gradient force	<1	3 DOF (3D)	Independent control	No	2		
	Gardi *et al.* [24]	Magnetic	Rotating	<1	2 DOF (2D)	Self-organization	Yes	Collective level	Fluid-air interface (2D)	Rotating, oscillating, forming chains
	Dong and Sitti [25]	Magnetic	Gradient force	∼80	2 DOF (2D)	Encode magnetic potential distributions	Yes	Collective level	Fluid-air interface (2D)	Formation of 2D pre-programmed shapes
	This work^d^	Magnetic	Swimming	**∼20**	**6 DOF** (3D)^c^	Magnetic coordination	Yes	Collective level	Underwater **(3D)**	**Dispersal, aggregation**, morphology regulation and locomotion
Macro scale	Rubenstein *et al.* [28]	On-board power	Vibrating	∼0.2	3 DOF (2D)	Active explicit coordination	Yes	Collective level	Land (2D)	Formation of complex shapes
	Zhou *et al.* [27]	On-board power	Flying	**∼20**	**6 DOF** (3D)	Active explicit coordination	Yes	∼10	Air (3D)	Field obstacle avoidance
	Berlinger *et al.* [26]	On-board power	Swimming	∼1	3 DOF (3D)	Active implicit coordination	Yes	∼10	Underwater (3D)	Dispersion, aggregation, dynamic circle formation

a BL, body length. ^b^Using bacteria as a power source has uncontrollable risks. ^c^6 DOF (constrained): our robot can pitch, yaw, roll, horizontally and vertically translate, and swim forwards, but cannot swim backwards. ^d^Move only in the gel to avoid the effects of gradient forces and gravity.

## RESULTS

### Fish-like constrained 6-DOF swimming of high-throughput fabricated robots

**Figure 2.**
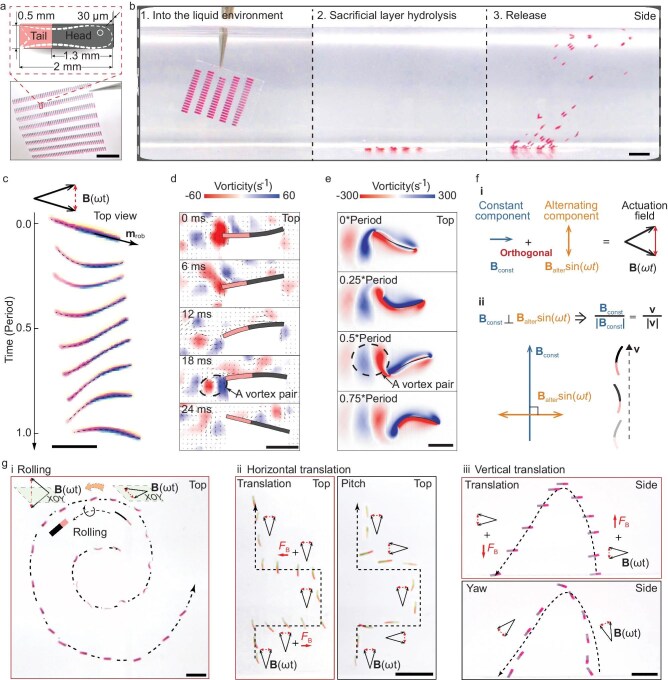
Fish-like constrained 6-DOF swimming of high-throughput fabricated robots. (a) The synchronized release robot array. Scale bar, 10 mm. (b) Release of the numerous robots. Scale bar, 10 mm. (c) The motion of a robot over a single motion cycle actuated by an oscillating magnetic field. Scale bar, 1 mm. (d) Experiment and (e) simulation results of the swimming robot. The vorticity field (color map) and velocity vector field (black arrows) are recorded during a motion cycle. The robot’s tail flaps backward against the water for propulsion, forming the reverse Karman vortex street. Scale bars, 1 mm. (f) The oscillating magnetic field is a superposition of a constant magnetic field **B**_const_ and an alternating magnetic field **B**_alter_sin(*ωt*). When the two components are orthogonal, the constant component **B**_const_ is in the same direction as the robot’s swimming direction. (g) More DOF. (i) Beyond the basic constrained 3 DOF (pitch, yaw and forward swimming), the robot can adjust its swimming posture via axial rolling. Additionally, it can perform translational movements (ii) horizontally and (iii) vertically, without necessitating adjustments in pitch or yaw. In total, the robot achieves up to six constrained DOF. Scale bars, 2 mm.

The fish-like magnetic soft robot features a rectangular film with a hard-magnetic elastomer head and silicone tail (Fig. 2a). Following size optimization, final dimensions are 2 × 0.03 × 0.5 mm^3^, with a 13 : 7 head-to-tail ratio. Fabricated via laser manufacturing, robots are interconnected by polyvinyl alcohol (PVA) films for synchronized release. The flexible PVA layer can be rolled or folded (Fig. S5) for transport and hydrolyzes within 5 min in water, releasing the robot swarm actuated by magnetic field (Fig. 2b, Movie S1).

An oscillating magnetic field **B**(*ωt*) actuates the robot’s head, causing passive tail flapping that mimics fish-like swimming (Fig. 2c, Movie S2) and generates forward thrust [29]. Experiments (Fig. 2d) and simulations (Fig. 2e) reveal vortex pair shedding at the tail, creating reverse von Karman vortex street wakes similar to fish [30,31]. Moreover, the robot’s force dynamics (Fig. S10) parallel those in swimming aquatic organisms [32], showing the characteristic double reversal of net force direction during each motion cycle.

The oscillating magnetic field is formed by the superposition of a constant magnetic field **B**_const_ and an alternating magnetic field **B**_alter_sin(*ωt*). These two magnetic field components are typically orthogonal [2,4], ensuring that the robot’s swimming direction aligns with the constant component (Fig. 2f). By adjusting the direction of the constant component, the pitch and yaw angles of the robot can be altered, thereby controlling its swimming direction. Furthermore, by rotating the alternating component **B**_alter_sin(*ωt*) around the constant component **B**_const_ of the oscillating magnetic field **B**(*ωt*), the thin-film robot can also roll around the magnetization direction, aligning its vibration orientation with the alternating component for the balance of the magnetic torque and the damping force (Fig. 2g i, Movie S3 Section 1). Compared to existing methods for rotation around the net magnetic moment [23,33], this approach is suitable for robot swarms, requiring lower design and control demands. Additionally, under the influence of a gradient magnetic field, the robot experiences magnetic forces **F**_B_, enabling additional horizontal (Fig. 2g ii) and vertical (Fig. 2g iii) translations without angular adjustments (Movie S3 Section 2). The robot possesses 3 rotational DOF (pitch, roll and yaw), 2 translational DOF (horizontal and vertical) and 1 constrained translational DOF (forward swimming), totaling 6 DOF (constrained).

### Maneuverability and applications of miniature swimming robots with constrained 6-DOF

**Figure 3.**
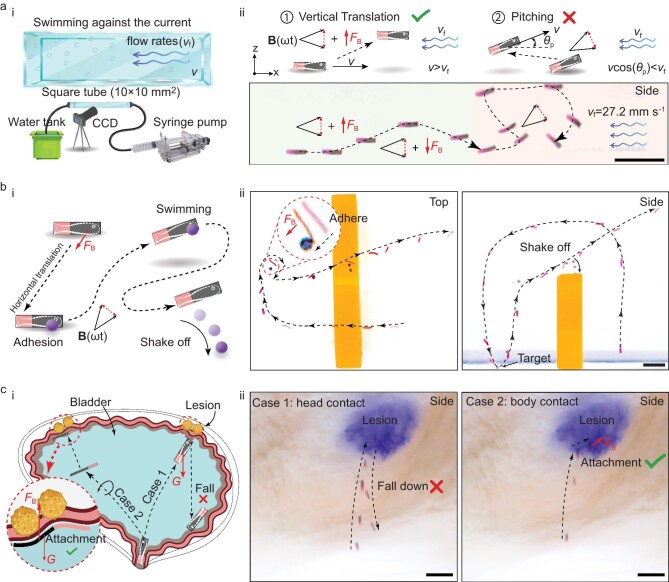
Maneuverability and applications of miniature swimming robots with more DOF. (a) The miniature robot swims against the flow. (i) Schematic of the experimental setup. (ii) Vertical translation allows the robots to rise and sink while resisting the flow (*v*_f_ = 27.2 mm s^−1^). Yet, the alteration of the pitch angle *θ*_p_ resulted in reduced forward velocity and potential failure against the flow. (b) 3D object transportation. The schematic (i) and experiment (ii) show that the robot can capture a polymethyl methacrylate (PMMA) sphere (0.5 mm) with adhesive force and release it at the target location by shaking motions. (c) The robot can attach itself to the lesion. The schematic (i) and experiment (ii) show that under gradient forces the robot moves horizontally and firmly adheres to the lesion (indicated with blue dye) in the bladder. All scale bars, 5 mm.

The miniature swimming robot with constrained 6-DOF has better maneuverability. First, employing a vision-based closed-loop control, the robot can precisely track pre-planned trajectories (Movie S4 Section 1) in both 2D and 3D (Fig. S11b and S11c). Second, horizontal and vertical translations enable the robot to resist gravity and ascend through vertical translation when forward propulsion is insufficient (Fig. S12, Movie S4 Section 2). What’s more, while swimming upstream, the robot can adjust its position through translational movement to avoid failure in upstream navigation due to the reduced velocity component against the flow caused by pitch or yaw adjustments (Fig. 3a, Movie S4 Section 3).

Additionally, the robot’s constrained 6-DOF capabilities enable tasks like 3D object transport (Fig. 3b, Movie S4 Section 4). The robot made close contact with the object using its body side to capture it via adhesion. Upon reaching the destination, an increased propulsion magnetic field induced greater vibrational amplitude, allowing the robot to release the object (Fig. S13). The robot can also serve as an efficient, non-invasive bioadhesive platform, attaching precisely to lesions within the bladder *ex vivo* to enhance healing (Fig. 3c, Movie S4 Section 5). Rather than touching lesion sites at the front end, the robot horizontally translated to make side contact with the sites and adhere tightly under the gradient force, maintaining attachment even after removing the gradient force.

### Differential directional regulation of robot individuals under global magnetic field actuation

The small size of robotic individuals limits their ability to deliver effective drug payloads, necessitating the formation of swarms to overcome individual limitations. Based on the feature that robot swimming direction aligns with the constant component **B**_const_ under orthogonal constant and alternating field conditions, programming spatial distribution patterns of the constant component **B**_const_ across the workspace can differentially regulate individual swimming directions, thereby inducing collective behaviors (Fig. S14).

**Figure 4.**
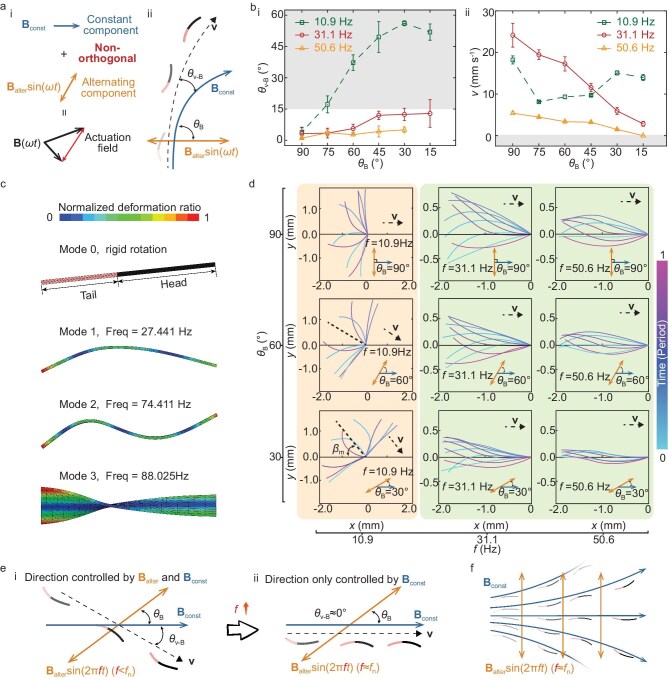
Differential individual directional regulation under global magnetic field actuation. (a) The non-90° angle *θ*_B_ between the constant and alternating components results in the robot’s swimming deviation angle *θ*_v-B_. (b) The relationship between *θ*_B_ and *f* with (i) the deviation angle *θ*_v-B_ between the speed and the constant component and (ii) robot speed *v* is recorded in experiments. Impractical combinations (*v* = 0 or *θ*_v-B_ > 15°) are indicated by gray colors. (c) Wet modal analysis. (d) Midline analysis. The robot’s midlines over a motion cycle were extracted. The snout tips of all midlines are aligned together to the coordinate origin for the convenience of motion direction analysis. The symmetry axis of the midlines’ envelope region is indicated by the black line with an angle *β*_m_ against the direction of constant components. The swimming direction of the robot aligns with this symmetry axis. (e) Complete control of swimming direction by the constant component. The angle *θ*_v-B_ tends to zero as the frequency *f* approaches the robot’s natural frequency *f*_n_ in liquid, which implies that the swimming direction of the robot is consistent with the constant component. (f) The spatially programmed constant component directs the swimming direction of each robotic agent within the workspace.

However, generating alternating field components that maintain perfect orthogonality to non-uniform constant field components is virtually impossible. Non-90° angles (*θ*_B_) between the constant component **B**_const_ and alternating component **B**_alter_sin(2π*ft*) result in undesired deviation angles (*θ*_v-B_) between velocity and the constant field direction (Fig. 4a). Fortunately, our parametric experiments (Fig. 4b i) reveal that as the frequency (*f*) of the alternating component **B**_alter_sin(2π*ft*) increases, deviations of the angle (*θ*_B_) from 90° progressively cease to affect the deviation angle (*θ*_v-B_).

This feature stems from the differential vibrational response of the soft robotic sheet structure to input excitations at varying frequencies. Using the Complex Orthogonal Decomposition (COD) method, the robot’s average traveling index was determined to be 0.2105, indicating significant standing wave components in its vibrational response. Therefore, based on modal superposition analysis theory, the robot’s vibrational response can be described as a linear combination of the dominant standing wave mode near the excitation frequency and other standing wave modes [34].

Wet modal simulations were used to determine the robot’s natural frequencies in water and the corresponding vibration modes (Fig. 4c). The results show that at lower frequencies (10.9 Hz), which are well below the first natural frequency (27.441 Hz), the robot’s response to the actuation magnetic field manifests primarily as rigid body rotation. At this stage, the angle (*θ*_B_) between alternating and constant components affects the robot’s orientation, leading to deviation (*θ*_v-B_) between swimming direction and the constant component direction. However, when the frequency (31.1 or 50.6 Hz) approaches or exceeds the first natural frequency, the robot’s response transitions from rigid rotation to vibrational deformation. At this point, the direction of alternating excitation only affects energy transfer efficiency without influencing the robot’s spatial orientation that determines swimming direction. The robot’s midline extracted from parametric experiments confirms that experimental results align with theoretical analysis (Fig. 4d).

Therefore, when the frequency (*f*) approaches the first natural frequency (*f*_n_) of the robot in fluid, the swimming direction is completely controlled by the constant magnetic field component **B**_const_, independent of the orthogonality between constant and alternating components (Fig. 4e). Since the robot operates at Reynolds numbers of approximately 20 to 60, it exhibits very rapid start–stop speeds (Fig. S15), allowing rigid body dynamics to be neglected. Leveraging this characteristic, under a uniform alternating magnetic field (*f ∼ f*_n_), the spatial distribution of the constant component **B**_const_ can be programmed to differentially guide the swimming direction of each robot within the workspace, thereby inducing collective behaviors (Fig. 4f). However, increasing frequency enhances damping forces acting on the robot, reducing both vibration amplitude (Fig. S16) and swimming speed (Fig. 4b ii). Consequently, the frequency for swarm coordination was determined to be 31.1 Hz.

Additionally, decreasing *θ*_B_ also leads to reduced swimming speeds (Fig. 4b ii). As *θ*_B_ decreases, the component of **B**_alter_sin(*ωt*) perpendicular to the robot’s long axis also decreases (Fig. S17), reducing energy transfer efficiency. This results in lower vibration amplitude of the tail *A*_T_ (Fig. S16), consequently decreasing swimming speed. Lower speeds increase sensitivity to environmental noise, such as hydrodynamic disturbances or undesired gradient forces caused by magnetic field non-uniformities, leading to directional deviations (Fig. 4b i). Therefore, during swarm coordination, alternating magnetic field components with high magnetic flux density are required to provide sufficient propulsion (see details in Text S12). Furthermore, the magnetic flux density of the constant magnetic field component should not exceed that of the alternating component to avoid speed reduction (see details in Text S13).

### Inter-individual distance regulation

**Figure 5.**
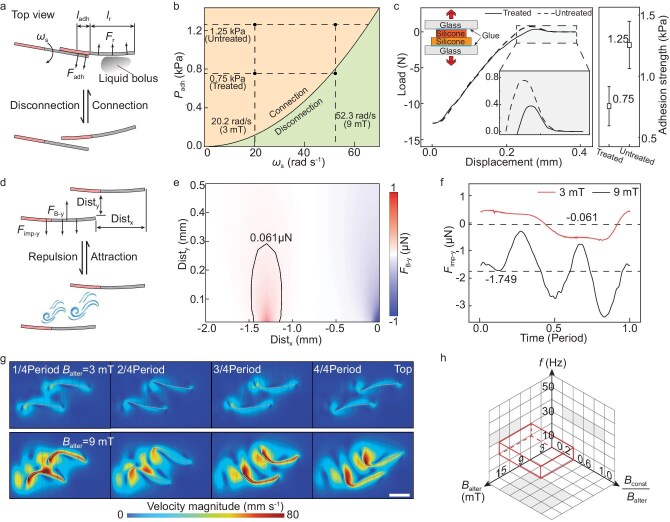
Inter-individual distance regulation. (a) Schematic of the forces that determine the connectivity of two robots. Disregarding magnetic interactions, the bond between the robots is governed by surface adhesion force (*F*_adh_) and fluid resistance (*F*_r_). *l*_r_ and *l*_adh_, respectively, represent the regions where fluid resistance and adhesive forces are applied. (b) Influence of adhesion strength (*P*_adh_) and rotational speed (*ω*_s_) on the connectivity status. Adjusting the norm of the alternating magnetic field component’s flux density amplitude (*B*_alter_) allows for changes in rotational speed, enabling control over the robots’ connection and separation. (c) Comparison of the adhesive forces of silicone surfaces treated with fetal bovine serum with untreated surfaces. The embedded schematic illustrates the test setup. The result indicates a halved surface adhesion for serum-treated silicone. Error bars indicate SD (*n* = 3). (d) The schematic diagram illustrates the forces that determine the repulsion or attraction between two robots. Dist_x_ and Dist_y_ denote the *x*- and *y*-distances between robots. The magnitude of the fluid impact pressure and magnetic force in the *y*-direction (*F*_imp-y_ and *F*_B-y_) determines whether the two robots attract or repel each other. (e) The simulation result shows the relationship between the distance of the two robots and the magnetic force acting on the robot. When *F*_B-y_ is positive, the two robots attract each other. (f) Simulated values of lateral impact pressure *F*_imp-y_ on two robots at *B*_alter_ = 3 or 9 mT during a motion cycle. When *F*_imp-y_ is negative, the two robots repel each other. At *B*_alter_ = 3 and 9 mT, the average *F*_imp-y_ on a single robot over one cycle is −0.061 μN and −1.749 μN, respectively. (g) The simulation result of the velocity magnitude (colored map) and velocity vector field (black arrows) around two robots during a motion cycle at *B*_alter_ = 3 and 9 mT. A larger alternating component results in faster fluid flow and greater impact pressure. Scale bar, 1 mm. (h) Feasible domains of magnetic field parameters for swarm coordination (spaces highlighted by red lines).

In swarms, surface adhesion between robotic agents disrupts collective behaviors by forming unwanted connections. These connections must be prevented to maintain robot independence. Two robots typically contact laterally (Fig. 5a), with the rear robot’s head aligning with the junction of the front robot’s head and tail through surface adhesion forces (*F*_adh_). Fluid resistance (*F*_r_) during oscillation at angular velocity (*ω*_s_) can counteract this adhesion and lead to disconnection. Our model analyzes how adhesion strength (*P*_adh_) and angular velocity (*ω*_s_) influence these connections (Fig. 5b). Increasing *ω*_s_ by enhancing the alternating component’s magnetic flux density amplitude (*B*_alter_) facilitates disconnection. However, higher *P*_adh_ requires greater *ω*_s_ to achieve disconnection, imposing higher demands on *B*_alter_. Consequently, we treated the robots with fetal bovine serum to reduce *P*_adh_ to an appropriate range (Fig. 5c).

After disconnection, robots should maintain distance from each other to prevent reconnection. The movement tendencies (attraction or repulsion) between nearby robots are primarily determined by magnetic forces (*F*_B_) and fluid impact pressure (*F*_imp_) (Fig. 5d). Simulation of the magnetic attraction (Fig. 5e) and fluid repulsion (Fig. 5f and g) between two robots shows that in a weak alternating magnetic field (*B*_alter_), the flow field around the robots is slower, resulting in lower impact pressure. This leads to a situation where, in certain regions, the fluid repulsion is weaker than the magnetic attraction, causing the robots to attract each other. Therefore, to prevent robots from approaching each other, a higher *B*_alter_ value must be employed.

In conclusion, based on the study of individual differential directional control and inter-individual distance regulation under a global magnetic field, we have experimentally determined a feasible domain of magnetic field parameters essential for swarm coordination (Fig. 5h). Predicated on these experimentally validated parameters, we will demonstrate the spatial collective behavior of robotic swarms, by programming the spatial distribution of the constant magnetic field components, to achieve lesion targeting and shape-adaptive adhesion.

### Active dispersal and aggregation behavior of the swarm for enrichment toward the lesion

**Figure 6.**
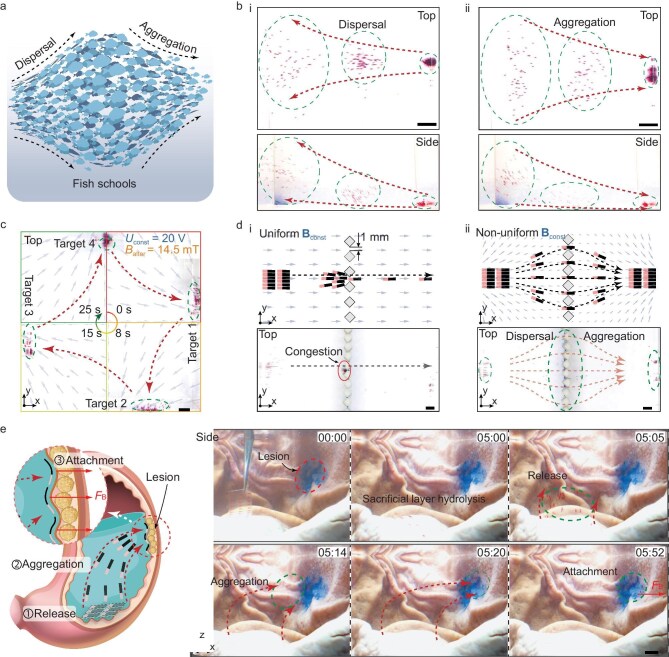
Active dispersal and aggregation behavior of the swarm for enrichment toward the lesion. (a) Schematic of the dispersal and aggregation of fish schools. (b) The robot swarm swims along the programmed constant magnetic field component, exhibiting 3D (i) dispersal and (ii) aggregation behaviors like the small fish school in (a). (c) A robot swarm follows the magnetic field for sequential aggregation at multiple target positions. (d) Navigation through the obstacle array. In contrast to (i) the congestion of the robot swarm under the uniform constant component **B**_const_, (ii) dispersed robot swarm under non-uniform **B**_const_ can pass through gaps. (e) *Ex vivo* demonstration of a robot swarm detaching from a PVA substrate, aggregating, and adhering to a gastric lesion. All scale bars, 10 mm.

The constant component (**B**_const_) of the oscillating magnetic field, which entirely controls the individual swimming directions without the requirement of orthogonality as the actuation frequency approaches the first-order natural frequency, can guide the robot swarm. By modulating the divergence and convergence of the constant component, the swarm can disperse and aggregate like a fish school (Fig. 6a). We designed a magnetic environment with a uniform alternating magnetic field component and a highly non-uniform constant magnetic field component. As shown in Fig. S22a, all Coil 2s were dynamically excited, producing a uniform alternating magnetic field component across the workspace. In contrast, only a pair of adjacent Coil 1s received constant current excitation, creating a highly non-uniform constant one. Given the non-uniformity of the constant magnetic field component across the workspace, we utilized the excitation voltage *U*_const_ on Coil 1 as an indirect reflection of the constant component’s magnetic flux density. By alternating the direction of the constant component (Fig. S22b), we can steer the robot swarm to switch between 3D dispersal and aggregation (Fig. 6b, Movie S5 Section 1). In addition, guided by the magnetic field, the robot swarm can also aggregate sequentially at multi-target locations (Fig. 6c, Movie S5 Section 2), or first disperse and then aggregate (Fig. S22c, Movie S5 Section 3).

The dispersal and aggregation empower the robot swarm with adaptability to complex environments. For example, the swarm can disperse to navigate through narrow gaps within arrays, thus avoiding congestion (Fig. 6d, Movie S6 Section 1). Alternatively, the robots can aggregate to pass through a singular passage (Fig. S23, Movie S6 Section 2). Moreover, an *ex vivo* experiment demonstrated effective lesion coverage by the aggregated swarm under gradient forces, highlighting its potential for drug delivery. As illustrated in Fig. 6e, after the deployment of the SyncRelease miniature robot array into the stomach, the PVA hydrolyzed within 5 min to release the robot swarm. The robot swarm aggregated along the converging magnetic field to the lesion site and adhered to it under gradient forces (Movie S7). In future clinical applications, it is feasible to coat the surfaces of robots with drug components without affecting their swimming capabilities. The robot can carry thermosensitive liposomes mixed with soft magnetic particles to achieve controlled drug release using high-frequency magnetic field heating [35].

### Morphology regulation and locomotion of swarm on the interface for better lesion coverage

**Figure 7.**
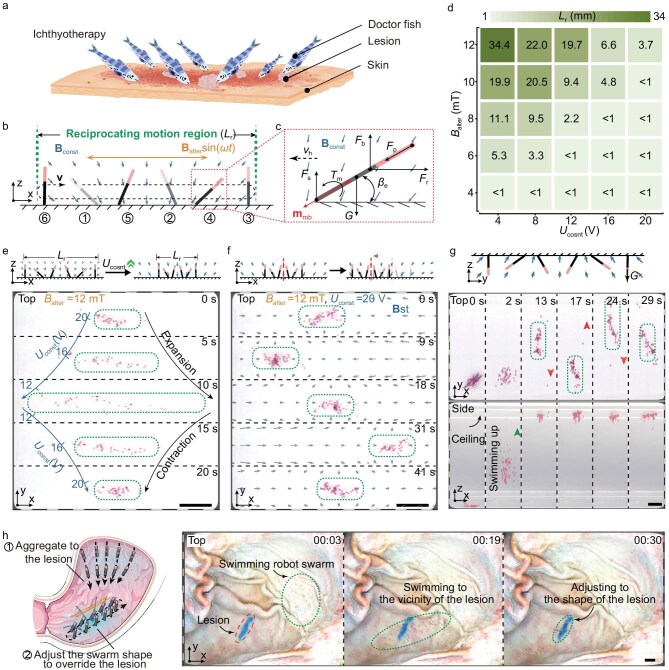
Morphology regulation and locomotion of swarm on the interface for better lesion coverage. (a) The doctor fish swarm, well-known for the treatment of skin conditions, is emulated in the work. (b) Schematic of the robot’s reciprocating motion within a motion range *L*_r_ (from position 1 to 6) on a solid–liquid interface under magnetic field and interface confinement. (c) Force diagram of the robot, subject to propelling force (*F*_p_), gravity (*G*), buoyancy (*F*_b_), fluid resistance (*F*_r_), supporting force (*F*_s_) and magnetic torque (*T*_m_), with the robot’s magnetic moment (**m**_rob_), horizontal speed (*v*_h_) and angle with the interface (*β*_e_) annotated. (d) Experimental results of the influence of alternating component flux density’s norm (*B*_alter_) and constant excitation voltage (*U*_const_) on the length *L*_r_. (e) The swarm morphology can be well regulated by varying the constant excitation voltage (*U*_const_). (f) The swarm locomotion is enabled by moving the convergence point of the constant magnetic field. (g) The robot swarm can form and adjust its position on the ceiling surface, overcoming gravity. (h) *Ex vivo* experiment showing the aggregation and morphology regulation of swarm for better coverage and drug delivery like the doctor fish swarm. All scale bars, 10 mm.

Upon reaching the interface where the lesion is located, the robot swarm can regulate its morphology and locomotion on the interface, similar to doctor fish schools during treatment (Fig. 7a). By planning the excitation of each coil (Fig. S24a), we generated a composite oscillating magnetic field with a uniform alternating component **B**_alter_sin(*ωt*) and a constant component **B**_const_ converging towards the interface (Fig. S24b). Under the action of multiple forces (Fig. 7c), the robotic agent reciprocates within a region of length *L*_r_, as if in a fishpen (Fig. 7b, Fig. S24c, Movie S8 Section 1). Analyzing the midlines of the robot at several positions (Fig. S24d), we found that the center of the reciprocating motion region is located at the convergence point of the constant magnetic field component. We developed a kinetic model for the reciprocating motion of individual robots (see details in Text S17) and measured the impact of the norm of the alternating magnetic field component’s flux density amplitude (*B*_alter_) and the constant excitation voltage (*U*_const_) on the length (*L*_r_) of the region (Fig. 7d). The model agrees well with experimental results, indicating that *L*_r_ can be extended by either increasing *B*_alter_ or decreasing *U*_const_.

With the addition of numerous robots, they are confined to reciprocating within a region centered around the convergence point of the constant component, forming a swarm of length *L*_r_. To ensure high propulsion, we maintained a high *B*_alter_ while adjusting *U*_const_ to regulate the swarm morphology. As depicted in Fig. 7e (Movie S8 Section 2), the swarm on the interface expanded and then contracted as *U*_const_ was first decreased and then increased. Additionally, by overlaying a uniform horizontal constant magnetic field, the convergence point of the constant magnetic field component can be changed (Fig. S26a), thus moving the reciprocating motion region to control swarm locomotion on the interface (Fig. 7f, Movie S8 Section 3). The robot swarm can regulate its morphology and locomotion (Fig. S26b, Movie S8 Section 4) for larger object transportation (Movie S8 Section 5). As shown in Fig. S27, the swarm contracted to grasp an object like a claw, moved it to the desired location, and then expanded to release the object.

The strong alternating component of the oscillating field provides sufficient propulsion for the swarm to adapt to complex environments, such as adjusting positions on inclined planes (Fig. S28a and S28b, Movie S9 Section 1), and even against gravity on the ceiling (Fig. 7g, Movie S9 Section 2). Robotic swarms can be imaged using ultrasonic technology. We simulated the turbid, non-homogeneous liquid environments potentially present in the body with a dilute starch solution and employed ultrasound (E2, SonoScape, Inc.) to image the swarm (Fig. S29a). Given the low starch content, the robots’ motion is considered unaffected. Under ultrasonic imaging, the swarm can be controlled to move within an opaque liquid (Fig. S29b, Movie S10).

The *ex vivo* experiment demonstrated the practical medical application of the robot swarm (Fig. 7h). The robot swarm first aggregated at a gastric lesion along the converging constant magnetic field component. Subsequently, the size and position of the reciprocating motion region are adjusted to regulate the swarm morphology and locomotion to better fit the lesion’s contour. Specifically, the swarm first converged into a linear formation under magnetic field confinement. The confining magnetic field was then rotated 90° around the interface normal, anchoring the swarm at the target location. Finally, the robot swarm firmly adhered to the lesion surface under the gradient magnetic field (Movie S11). Additionally, as the robots scrape against the interface during reciprocating motion, the swarm has the potential to be used to clear biofilm eradication from the body [36].

## DISCUSSION

We have realized a miniature magnetic soft robot swarm capable of complex fish-like collective swimming behaviors through individual differential direction control and inter-individual distance regulation. The individual robots are constructed from hard-magnetic elastomers with heterogeneous head and tail material. They can be fabricated through a high-throughput process. Under the actuation of oscillating and gradient magnetic fields, the proposed robotic agent achieves constrained 6-DOF in motion (pitch, yaw, roll, horizontal and vertical translation, and forward swimming, unable to swim backward). We further found experimentally that when the frequency of the oscillating magnetic field approaches the natural frequency of the robot in the liquid, the swimming direction of the robot is not influenced by the angle between the constant and alternating components of the oscillating field, but is solely governed by the constant component. Thus, robots across the workspace can swim along the path patterns determined by the constant component. Additionally, the inter-robot distance can be coordinated by fluidic repulsive force under an alternating component with suitable strength. Through dynamically programming the spatial distribution patterns of the constant component and fine-tuning the strength of the uniform alternating component, the robot swarm can disperse and aggregate in 3D aquatic environments like migrating fish schools in the swimway. The swarm can also be controlled by the magnetic field and interface confinement to regulate its morphology and locomotion on the interface like foraging fish schools in a fishpen. This robot swarm can be used to target and accumulate at the lesion site, adapting to its shape for effective drug delivery.

Our work offers insights and guidance for steering swarm formation through external magnetic fields as confinement. The confinement does not necessarily imply a tangible boundary (e.g. a membrane). Here, we steered the emergence of collective behavior by confining the swimming directions of individuals with an intangible magnetic field. Increasing the number of electromagnets [37] or using mobile ones [38] can not only enrich the constant component’s spatial distribution to foster the emergence of more complex swarm behaviors, but also reduce the occurrence of singularities [39]. Additional confinement methods, such as acoustic fields [40] or flexible boundaries [41], may induce new behaviors.

Adjusting the surface adhesion strength of the fish-like magnetic soft robot according to specific tasks is crucial. For instance, high adhesion is required for individual robots to perform 3D transport tasks, while moderate adhesion facilitates stable swimming and easy separation of assembled robots. For robot swarms, lower adhesion prevents sticking-induced swimming failures, whereas high adhesion is essential for anchoring at lesion sites against fluid washout. Therefore, biocompatible materials with adjustable adhesion strength can be employed for surface modification as needed [42].

Future clinical studies should focus on the delivery and retrieval of robot swarms. The synchronized release robot array can be designed as an origami structure [43] that can actively unfold and release the robot swarm after being delivered into the body by a continuum robot [44,45]. Alternatively, continuum robots can directly deliver the swarm, reducing deployment time. This approach effectively guards against the individual robots in the swarm being swept away and lost en route due to the forces within the complex fluidic environments [46]. After the completion of missions, the robot swarm will aggregate at a designated location and be retrieved by the continuum robot (Fig. S30, Movie S12). Additionally, the design of therapeutic payloads for this robotic platform requires careful consideration of drug–material interactions and release kinetics. Various drug loading strategies can be implemented, including physical adsorption onto the robot surface, encapsulation within the polymer matrix during fabrication, or chemical conjugation to functional groups on the robot exterior. For example, biodegradable soft materials [47] can be considered as a replacement for silicone. The degradable robot swarm, which is attached to the lesion, will slowly degrade and release medication, while the remaining biocompatible SiO_2_-coated neodymium–iron–boron (NdFeB) microparticles [48] will be expelled from the body via metabolic processes. We anticipate that, supported by advanced imaging technologies, our robot swarm will be used for the safe and efficient delivery of therapeutic payloads in the clinic.

## MATERIALS AND METHODS

### Preparation processes

Fish-like magnetic soft robots were fabricated using commercially available materials including 00-30 silicone, particles, NdFeB and PVA, with biocompatibility achieved through silica coating of magnetic particles via the Stöber method to reduce biotoxicity [49]. The mass production process employed laser fabrication technology (Fig. S4), beginning with spin-coating of PVA films onto plasma-cleaned ceramic substrates, followed by casting and curing of silicone within 3D-printed frames to form structured arrays through selective laser cutting and removal of alternating strips, subsequent filling of gaps with magnetic particle-infused silicone composite (5 : 1 w/w ratio), precision cutting of robot arrays using a 355 nm UV laser, longitudinal magnetization via 5 T pulsed magnetic field, and final detachment facilitated by vacuum heating at 100°C, which reduces the PVA–substrate contact area through ceramic micropore gas expansion (see details in Text S1).

### Experimental setup

A magnetic actuation system (Fig. S7) was developed for independent and precise programming of constant and alternating magnetic [50] fields through superposition of **B**_const_ and **B**_alter_sin(*ωt*) components to generate composite actuation fields **B**(*ωt*) (Fig. S6). The system comprised eight electromagnets arranged in pairs at hexahedral vertices, each containing dual coils excited by power amplifiers for DC and AC components, respectively, with series resonant circuits compensating inductive reactance at 31.1 Hz operating frequency. Custom LabVIEW software integrated real-time imaging tracking and control algorithms dispatching signals via the CompactDAQ system, enabling rapid calculation of control signals from target magnetic fields assuming linear superposition. The apparatus generates magnetic flux densities up to 43 mT omnidirectionally with alternating frequencies reaching 100 Hz, incorporates a 200 × 200 × 200 mm^3^ transparent water tank workspace, dual orthogonal CCDs for trajectory capture, a three-axis loading platform for clinical investigation compatibility, and real-time Hall sensor monitoring with serial feedback for system error correction including temperature-induced resistance changes (see details in Text S1).

### Swimming simulation of the robot

We utilized commercial finite element software (COMSOL) for the 2D fluid–structure interaction simulation of robotic swimming. Initially, based on extracted midline data (Fig. S9a), we established the transversal displacement equation for the robot *y*(*X, t*) (Fig. S9b). The amplitude of this transversal displacement equation was applied to the robot’s neutral plane to simulate its actual motion posture. In an unconstrained free state, the robot advances forward through interaction with the fluid. Dynamic meshing and mesh reconstruction techniques were employed to enhance the accuracy and convergence of the simulation. The flow field surrounding the robot and the forces exerted on the robot in a liquid environment were extracted for analysis. The same methodology was applied when simulating the interaction of two closely approaching robots with the fluid. The lateral impact pressure exerted on the two robots was extracted for comparison with the magnetic force. Detailed simulation parameters are provided in Table S4.

## Supplementary Material

nwaf429_Supplemental_Files

## Data Availability

All data are available in the main text or the supplementary materials. Additional data related to this paper can be requested from the authors.
