# Supplementary material for: Swarm coordination of fish-like magnetic soft robots: directed aggregation and shape-adaptive attachment toward efficient drug delivery
Source: Natl Sci Rev. 2025 Oct 21;12(12):nwaf429. doi: 10.1093/nsr/nwaf429 (PMC12628758; doi:10.1093/nsr/nwaf429)
Supplement: nwaf429_Supplemental_Files [file nwaf429_supplemental_files.zip › 2 Supplementary information.pdf]

Supplementary Information for

Swarm coordination of fish-like magnetic soft robots: directed aggregation and shape-adaptive attachment toward efficient drug delivery

Liyang Mao[1]†, Chenyao Tian[1]†, Peng Yang[1], Xianghe Meng[1]*,
Xingjian Shen[1], Hao Zhang[1]*, and Hui Xie[1]*

*Corresponding author. Xianghe Meng, mengxianghe@hit.edu.cn; Hao Zhang, haoz@hit.edu.cn;
Hui Xie, xiehui@hit.edu.cn

This PDF file includes:

Supplementary Text S1 to S17

Tables S1 to S4

Figs. S1 to S30

Legends for movies S1 to S12

Other Supplementary Materials for this manuscript include the following:

Movies S1 to S12

Supplementary Note 1 Supplementary Text.

S1. The detailed preparation processes

The fabrication of the fish-like magnetic soft robots utilizes commercially available materials, including 00-30 silicone (Smooth-On, Inc.), neodymium iron boron particles (5 μm, Magnequench Co. Ltd) and polyvinyl alcohol (Aladdin Co., Ltd.). Silicone is a material widely used in biomedicine. Silica can be coated on NdFeB particles using the Stöber method, thereby effectively reducing their biotoxicity. Our previous study [49] has fully demonstrated the feasibility of this method through cytotoxicity experiments. Thus the robots have potential for biocompatibility.

We used laser fabrication to produce robots in large quantities, which are interconnected by PVA films to form a synchronized release array (Fig. S4). Initially, a 10 % w/w PVA solution is uniformly applied to a silicate ceramic substrate, which has been cleansed with oxygen plasma (TS-SY05, Shenzhen Tonson Tech Automation Equipment Co., Ltd.), via a spin coater (KW-4A, Suzhou CChip scientific instrument Co., Ltd.). The substrate is left at room temperature to allow for the evaporation of water, resulting in a uniform PVA film formation.

Subsequently, a 30-μm-thick square frame, produced by 3D printing, is placed upon the PVA layer. Next, the A and B components of 00-30 silicone are mixed and degassed using a planetary mixer (SK-300SII, Shashin Kagaku Co., Ltd.). The red-dyed mixture is then poured into the square frame on top of the PVA film, leveled with a scraper, and allowed to cure at room temperature. After curing, a laser cuts the silicone into strips, and every alternate strip is peeled away, leaving a structured array of silicone strips. The gaps created by the peeling are filled with a mixture of 00-30 silicone and unmagnetized NdFeB particles (5:1 w/w), which is then allowed to cure at room temperature, forming a composite elastomeric layer with alternating stripes of pure silicone and magnetic particle-infused silicone.

Subsequently, a 355 nm ultraviolet laser precisely cuts the elastomeric layer to carve out the robot array as per design requirements. After removing the excess material, the robots are magnetized along their longitudinal axis using a 5 T pulsed magnetic field in a magnetizing machine (J3801 Nanjing Golden Wolf Electric Co.). Finally, the ceramic substrate with the robot array is placed in a 100 °C vacuum oven

(Shanghai Yiheng Instrument Co., Ltd.). The expansion of gases within the ceramic micropores reduces the contact area between the PVA film and the ceramic base, facilitating the effortless detachment.

S2. Impact of the dimensions on swimming velocity

We examined the impact of the robot's dimensions on its swimming velocity, using relative velocity (the ratio of absolute velocity to body length) for comparative analysis. It is observed that relative velocity initially increases with length and thickness but then decreases (Fig. S3a and b). This trend is attributed to the direct effect of these dimensions on the robot's natural frequency. As the natural frequency approaches the driving frequency of the magnetic field, the amplitude and consequently the velocity increase [52]. As depicted in Fig. S3c, the relative velocity positively correlates with the robot's height. An increase in height not only augments the volume of the magneto-elastic body to enhance the magnetic torque but also enlarges the tail's surface area to generate more propulsive forces. However, a substantial increase in height (close to or over the length) may alter the robot's vibrational modes, leading to locomotion inefficiencies. Fig. S3d indicates that the head-to-tail ratio does not significantly impact velocity. Nevertheless, a heterogeneous tail is essential to create a differential in amplitude between the head and tail, which is crucial for efficient propulsion. The standard dimensions of the robots used in all experiments, unless otherwise specified, are 2 mm in length, 30 μm in thickness, and 0.5 mm in height, with a head-to-tail length ratio of 13:7.

S3. PIV system

Particle Image Velocimetry (PIV) technology was employed to visualize the characteristics of fluid flow around the miniature fish-like magnetic soft robot. Uniformly dispersed in the experiment tank were 10 μm polystyrene particles (Beiting Measurement Technology Co., Ltd.). The laser beam (532 nm) was expanded into a 0.5 mm thick plane and projected along the plane of the robot's motion to illuminate the polystyrene particles. The movement of the particles surrounding the robot was captured at a rate of 1000 fps by a camera (acA2040-120um, Basler, Inc.), achieved by minimizing the exposure area. The recorded image sequences were then processed using the OpenPIV software [53], applying cross-correlation algorithms to analyze.

S4. Analysis of the midlines and transversal displacement

We employed a high-speed CCD (acA2040-120um, Basler, Inc.) to capture the cyclic motion postures of a miniature fish-like magnetic soft robot. By reducing the camera's exposure area, we were able to enhance the frame rate to 300 fps. For the extraction of the midlines from the captured data, we adopt the following procedures utilizing the OpenCV library: (i) The pre-processed images were subjected to binary thresholding. (ii) We detected the edges of the robot and performed a skeletonization process. (iii) The Gaussian filter was used to smooth the curve. (iv) Unless otherwise noted, the snout tips of all the extracted midlines were aligned together in the direction of motion.

Based on the midline data in an Euler coordinate system, the data for the transversal displacement of the axis at position X along the robot's body can be obtained through line integration. Using the swimming motion of fish as a reference, the transversal displacement $y(X,t)$ of the robot's axis can be defined as the product of the envelope Env(X) and the backward wave [54]. To facilitate convergence in the simulation, a startup function also needs to be introduced.

$$\begin{aligned} y(X,t) &= \mathrm{Env}(X)\sin(\gamma X + \omega t)\left(1-\exp\left(-t/t_a\right)\right) \\ \mathrm{Env}(X) &= \frac{k_1}{l_{rob}}X^2 + k_2 X + k_3 l_{rob} \end{aligned} \tag{S1}$$

where, γ is the wave number, $\omega=2\pi f$ is the robot's angular frequency, l_{rob} is the robot length, k_1, k_2, and k_3 are amplitude coefficients of the envelope Env(X), and t_{a} is the characteristic time for activation. At $t_{\mathrm{a}} = \infty$, the lateral displacement during stable swimming of the robot can be obtained.

S5. Complex modal decomposition

To investigate the proportion of the traveling wave and standing wave in the transversal displacement of the robot's axis, we employed the Complex Orthogonal Decomposition (COD) analysis method [54]. During a vibration cycle, the data for the transversal displacement of the robot's axis can be expressed as:

$$\begin{aligned} \mathbf{Y}_{M\times N} &= \left[\mathbf{y}_1, \mathbf{y}_2, \ldots, \mathbf{y}_M\right]^{\mathrm{T}} \\ \mathbf{y}_j &= [y_j(t_1), y_j(t_1), \ldots, y_j(t_N)] \end{aligned} \tag{S2}$$

where, the component $y_m(t_n)$ at row m and column n of matrix $\mathbf{Y}$ represents the transversal displacement $y(X_m,t_n)$ of the robot axis at point X_m (m = 1,2,..., M) and time t_n (n = 1,2,..., N). The points X_m are evenly spaced along the robot body, and the time instants t_n are also uniformly sampled within a vibration cycle. Using Hilbert transform, a complex matrix $\mathbf{Z}$ can be generated based on the matrix $\mathbf{Y}$:

$$\begin{aligned} \mathbf{Z}_{M\times N} &= \left[\mathbf{z}_1,\mathbf{z}_2,\ldots,\mathbf{z}_M\right]^T \\ \mathbf{Z}_m(t) &= \mathbf{y}_m(t) + iH(\mathbf{y}_m(t)) \end{aligned} \tag{S3}$$

where, $\mathbf{Z}_m(t)$ is the analytical vector of $\mathbf{y}_m$, i represents the imaginary unit and H means Hilbert transform. A complex correlation matrix $\mathbf{R}=\mathbf{Z}\overline{\mathbf{Z}}^{\mathrm{T}}/N$ is then generated, where the overbar indicates the conjugate of a complex number. The matrix $\mathbf{R}_{M\times M}$ is a complex Hermitian matrix, which has real eigenvalues λ_m and complex eigenvectors $\mathbf{w}_m$. The traveling index α is then calculated as:

$$\alpha = 1/\operatorname{cond}(\mathbf{W}) \tag{S4}$$

where, $\mathbf{W}$ = [real($\mathbf{w}$), imag($\mathbf{w}$)] is a matrix with its two columns being the real and imaginary part of $\mathbf{w}$, respectively, and cond($\mathbf{W}$) denotes the condition number of $\mathbf{W}$. The value of the traveling index is defined as the reciprocal of the relative condition number. If the traveling index is zero, the real and imaginary vectors are completely independent, indicating that the robot's transversal displacement can be represented by a pure standing wave. If the traveling index is 1.0, the two vectors are coherent, meaning that the transversal displacement can be represented by a pure traveling wave.

S6. Analysis of wet modal vibration

To investigate the vibration modes and corresponding frequencies of the proposed robot in a liquid environment, we conducted a wet modal vibration analysis using the commercial finite element software (Abaqus). The analysis considered the fluid-structure interaction (FSI). The geometric models for both the robot and its aquatic environment were constructed, with assigned material properties (**Table S4**). The robot's tail was made of silicone 00-30 and its head comprised silicone 00-30 mixed with NdFeB particles in a 5:1 mass ratio. The robot was simulated in a free state without external constraints. After defining the

FSI interface on the robot, the mesh was generated with refinement near the interaction surface. Finally, the analysis was set up and executed to obtain the wet modal vibration characteristics of the robot.

S7. Experimental setup

We developed a magnetic actuation system, enabling independent and precise programming of both constant and alternating magnetic fields. By superimposing the constant component $\mathbf{B}_{const}$ and alternating component $\mathbf{B}_{alter}\sin(\omega t)$, the desired composite actuation magnetic field $\mathbf{B}(\omega t)$ can be generated (Fig. S6). The physical setup of our magnetic actuation experiment is presented in Fig. S7. The equipment described herein utilized this apparatus, except for the trajectory tracking experiments, which used three pairs of orthogonally arranged Helmholtz coils. The magnetic actuation system comprised eight electromagnets arranged in pairs along the vertices of a regular hexahedron to generate composite magnetic fields. Each electromagnet was numbered for ease of reference and included a small coil (Coil 1), a larger coil (Coil 2), and a silicon steel core. Coils 1 and 2 were excited by power amplifiers (HEA-200C, Nanjing Foneng Technology Industry Co., Ltd. and ATA-300, Xi'an Aigtek Electronic Technology Co., Ltd.) for DC and AC excitation, respectively, to produce constant and alternating components. Series resonant circuits were implemented to compensate for inductive reactance at operating frequency (31.1 Hz), reducing impedance and power requirements [50]. A host computer was used to run a custom LabVIEW-based software, encompassing a real-time imaging tracking program and control algorithms, which dispatch control signals to the amplifiers via a CompactDAQ system (NI-9174, NI-9263, National Instruments, Inc.). Assuming linear superposition of the magnetic fields generated by each electromagnet, control signals can be quickly calculated from the target magnetic field (Text S8-S10). The actuation system can generate a magnetic flux density of up to 43 mT in any direction at the center position. The alternating magnetic field can reach frequencies of up to 100 Hz. A 200 × 200 × 200 mm transparent water tank as the experimental environment can be placed in the device without mechanical collisions. Two orthogonally positioned CCDs (acA2040-120um, Basler, Inc.) captured the swimming trajectory of the proposed robot under magnetic actuation. Additionally, a loading platform (three-axis electric translation stage) was introduced to maintain the

experimental tank within the ROI of the magnetic actuation system, ensuring a workspace suitable for potential clinical investigations. To monitor the drive magnetic field in real time during the experiment, a Hall sensor (TLE493D-W2B6, Infineon Technologies Inc.) was fixed on the loading platform. Its output signals were acquired via the serial port of the host computer, which served as feedback for adjusting system errors, such as resistance changes due to temperature increases.

S8. Modeling of the magnetic dipole

A dipole model was employed for the magnetic field modeling of individual electromagnets, as it offers an analytical expression convenient for computational purposes. The magnetic flux density **B** produced by a dipole can be given as [*55*]

$$\mathbf{B}(\mathbf{m}_{\mathrm{d}},\mathbf{r})=\frac{\mu_0}{4\pi}\left(\frac{3\mathbf{m}_{\mathrm{d}}\bullet\mathbf{r}}{|\mathbf{r}|^5}\mathbf{r}-\frac{\mathbf{m}_{\mathrm{d}}}{r^3}\right) \tag{S5}$$

where $\mu_0 = 4\pi \times 10^{-7}$ is the permeability of vacuum, the vector pointing from the dipole $\mathbf{P}_{\mathrm{m}}$ to the targeted point **P** is denoted as $\mathbf{r} = \mathbf{P} - \mathbf{P}_{\mathrm{m}}$, and $\mathbf{m}_{\mathrm{d}}$ is the magnetic moment of the dipole, proportional to the excitation voltage of the electromagnet. To calculate the parameters $\mathbf{P}_{\mathrm{m}}$ and $\mathbf{m}_{\mathrm{d}}$ of the dipole model for individual electromagnets, we collected magnetic flux density data within the workplace of the magnetic actuation system. A high-precision 3D Hall sensor (CH-3600, CH-Magnetoelectricity Technology Co.) (Fig. S8) was mounted on a 4-DOF platform and then extended into the workplace. To facilitate precise measurement of the magnetic flux density at any point within the workspace, LabVIEW-based software was developed for comprehensive traversal of the sensor throughout the workspace.

S9. Modeling of the magnetic force and torque

The miniature fish-like magnetic soft robot was designed with a magnetic moment along its longitudinal axis, allowing it to be remotely subjected to magnetic forces and/or torques in an external magnetic field. Within a magnetic field characterized by a flux density **B**, the robot was subjected to torque $\mathbf{T}_{\mathrm{m}}$. This torque aligned the robot's magnetic moment $\mathbf{m}_{\mathrm{rob}}$ with the direction of the magnetic field [*56*].

$$\mathbf{T}_{\mathrm{m}} = \mathbf{m}_{\mathtt{rob}} \times \mathbf{B} = \begin{bmatrix} 0 & B_z & -B_y \\ -B_z & 0 & B_x \\ B_y & -B_x & 0 \end{bmatrix} \begin{bmatrix} m_{\mathtt{rob-x}} \\ m_{\mathtt{rob-y}} \\ m_{\mathtt{rob-z}} \end{bmatrix} \tag{S6}$$

Additionally, the robot experienced a magnetic force, denoted as $\mathbf{F}_{\mathrm{B}}=(\mathbf{m}_{\mathrm{rob}}\bullet\nabla)\mathbf{B}$, when operating within a non-uniform magnetic field. Given that there was no current flowing through the space occupied by the object, the quasi-static magnetic field can be described by Maxwell's equations as satisfying $\nabla\times\mathbf{B}=0$ and $\nabla\bullet\mathbf{B}=0$. Consequently, the magnetic force $\mathbf{F}_{\mathrm{B}}$ acting on the robot at point **P** can be represented in a more intuitive form.

$$\mathbf{F}_{\mathrm{B}} = \begin{bmatrix} \frac{\partial \mathbf{B}(\mathbf{P})}{\partial x} & \frac{\partial \mathbf{B}(\mathbf{P})}{\partial y} & \frac{\partial \mathbf{B}(\mathbf{P})}{\partial z} \end{bmatrix}^{\mathrm{T}} \begin{bmatrix} m_{\mathtt{rob-x}} \\ m_{\mathtt{rob-y}} \\ m_{\mathtt{rob-z}} \end{bmatrix} \tag{S7}$$

S10. Programming methods of the composite magnetic field

The composite actuation magnetic field is formed by the superposition of constant and alternating magnetic fields. The magnetic actuation system consists of eight electromagnets. The eight electromagnets operate independently without mutual interference and function within their linear ranges. Consequently, the resultant total magnetic field conforms to the principle of superposition. Each electromagnet includes two coils, designated as Coil 1 and Coil 2 (Fig. S6). Coil 1, under constant excitation, generates a constant magnetic field. Coil 2, under alternative excitation, produces an alternating magnetic field.

Generation of the constant field by Coil 1

We directly control the constant field to navigate the robot, rather than the magnetic torque. Based on the magnetic dipole model, we can calculate the magnetic flux density of the constant field $\tilde{\mathbf{B}}_{\mathrm{const}-i}(\mathbf{P})$ at the target point **P**, generated by the *i*-st Coil 1 under a unit constant current excitation. The magnetic flux density of the overall constant field is obtained by linearly superposing the magnetic flux densities produced by each Coil 1. Furthermore, within the linear range, the magnetic flux density produced by the electromagnet is directly proportional to the excitation current. Thus, at point **P** in the workspace, the constant field's

magnetic flux density $\mathbf{B}_{\text{const}}(\mathbf{P})$, generated by driving eight Coil 1 with current $\mathbf{I}_{\text{const}} = [I_{\text{const-1}} \ldots I_{\text{const-}i}]$, can be represented as

$$\mathbf{B}_{\text{const}}(\mathbf{P}) = \left[\tilde{\mathbf{B}}_{\text{const}-1}(\mathbf{P}) \cdots \tilde{\mathbf{B}}_{\text{const}-i}(\mathbf{P}) \right] \begin{bmatrix} I_{\text{const-1}} \\ \vdots \\ I_{\text{const-}i} \end{bmatrix} = \tilde{\mathrm{B}}(\mathbf{P}) \mathbf{I}_{\text{const}} \tag{S8}$$

where, $\tilde{\mathrm{B}}(\boldsymbol{P})$ represents the contribution matrix of each Coil 1 to the magnetic flux density. When a pair of electromagnets (such as EM 1-1 and EM 1-2) are excited with currents of equal magnitude but opposite directions, this is considered to produce an approximately uniform magnetic field. Under this condition, the magnetic flux density at the central point **O** is regarded as representative of the constant field in the workspace. Therefore, the current $\mathbf{I}_{\text{const}}$ required to generate a uniform constant field with a magnetic flux density $\mathbf{B}_{\text{const}}(\mathbf{O})$ in the workspace can be calculated as follows:

$$\mathbf{I}_{\text{const}} = \tilde{\mathrm{B}}(\mathbf{O})^{\dagger} \mathbf{B}_{\text{orien}}(\mathbf{O}) \tag{S9}$$

where † denotes the pseudo-inverse of the matrix. The uniformity of the constant field allows for the simultaneous manipulation of robots throughout the entire workspace. If uniformity is not required, a force $\mathbf{F}_{\text{B}}$ can be applied to the robots through magnetic field gradients, providing them with DOFs for horizontal (left-right) and vertical (up-down) translation. However, this magnetic field configuration can accurately manipulate only the robot at a specific location at a time. At the target point **P**, the magnetic flux density gradient produced by *i*-st Coil 1 under a unit current excitation is denoted as $\nabla\tilde{\mathbf{B}}_{\text{const}-i}$. The gradient of magnetic flux density also follows the principle of superposition. Therefore, the constant field's magnetic flux density gradient $\nabla\mathbf{B}_{\text{const}}$ produced by driving eight Coil 1 with current $\mathbf{I}_{\text{const}}$ can be represented as:

$$\nabla\mathbf{B}_{\text{const}}(\mathrm{P}) = \begin{bmatrix} \dfrac{\partial\mathbf{B}_{\text{const}}(\mathrm{P})}{\partial x} & \dfrac{\partial\mathbf{B}_{\text{const}}(\mathrm{P})}{\partial y} & \dfrac{\partial\mathbf{B}_{\text{const}}(\mathrm{P})}{\partial z} \end{bmatrix}$$

$$\begin{aligned}
\frac{\partial\mathbf{B}_{\text{const}}(\mathbf{P})}{\partial x} &= \begin{bmatrix} \dfrac{\partial\tilde{\mathbf{B}}_{\text{const}-1}(\mathbf{P})}{\partial x} & \cdots & \dfrac{\partial\tilde{\mathbf{B}}_{\text{const}-i}(\mathbf{P})}{\partial x} \end{bmatrix} \begin{bmatrix} I_{\text{const-1}} \\ \vdots \\ I_{\text{const-}i} \end{bmatrix} = \widetilde{\mathsf{B}_{\partial x}}(\mathbf{P})\mathbf{I}_{\text{const}} \\
\frac{\partial\mathbf{B}_{\text{const}}(\mathbf{P})}{\partial y} &= \begin{bmatrix} \dfrac{\partial\tilde{\mathbf{B}}_{\text{const}-1}(\mathbf{P})}{\partial y} & \cdots & \dfrac{\partial\tilde{\mathbf{B}}_{\text{const}-i}(\mathbf{P})}{\partial y} \end{bmatrix} \begin{bmatrix} I_{\text{const-1}} \\ \vdots \\ I_{\text{const-}i} \end{bmatrix} = \widetilde{\mathsf{B}_{\partial y}}(\mathbf{P})\mathbf{I}_{\text{const}} \\
\frac{\partial\mathbf{B}_{\text{const}}(\mathbf{P})}{\partial z} &= \begin{bmatrix} \dfrac{\partial\tilde{\mathbf{B}}_{\text{const}-1}(\mathbf{P})}{\partial z} & \cdots & \dfrac{\partial\tilde{\mathbf{B}}_{\text{const}-i}(\mathbf{P})}{\partial z} \end{bmatrix} \begin{bmatrix} I_{\text{const-1}} \\ \vdots \\ I_{\text{const-}i} \end{bmatrix} = \widetilde{\mathsf{B}_{\partial z}}(\mathbf{P})\mathbf{I}_{\text{const}}
\end{aligned} \tag{S10}$$

where, $\widetilde{\mathsf{B}_{\partial x}}$, $\widetilde{\mathsf{B}_{\partial y}}$, and $\widetilde{\mathsf{B}_{\partial z}}$ represent the contribution matrices of each Coil 1 to the gradients of the magnetic flux density in the *x*, *y*, and *z* directions, respectively. According to Eq. (S7), the force $\mathbf{F}_{\mathrm{B}}$ exerted on a robot at position **P** can be represented as:

$$\mathbf{F}_{\mathrm{B}} = \begin{bmatrix} \dfrac{\partial\mathbf{B}_{\text{const}}(\mathbf{P})}{\partial x} & \dfrac{\partial\mathbf{B}_{\text{const}}(\mathbf{P})}{\partial y} & \dfrac{\partial\mathbf{B}_{\text{const}}(\mathbf{P})}{\partial z} \end{bmatrix}^{\mathrm{T}} \mathbf{m}_{\text{rob}} = \begin{bmatrix} \mathbf{m}_{\text{rob}}{}^{\mathrm{T}}\widetilde{\mathsf{B}_{\partial x}}(\mathbf{P}) \\ \mathbf{m}_{\text{rob}}{}^{\mathrm{T}}\widetilde{\mathsf{B}_{\partial y}}(\mathbf{P}) \\ \mathbf{m}_{\text{rob}}{}^{\mathrm{T}}\widetilde{\mathsf{B}_{\partial z}}(\mathbf{P}) \end{bmatrix} \mathbf{I}_{\text{const}} \tag{S11}$$

where, $\mathbf{m}_{\text{rob}}=\mathbf{M}_{\text{NdFeB}}\cdot V_{\text{NdFeB}}$ is the magnetic moment of the robot, $\mathbf{M}_{\text{NdFeB}}$ is the magnetization strength of NdFeB, and V_{NdFeB} is the volume of NdFeB contained in the robot. Based on Eq. (S8) and (S11), we can establish the relationship between the constant field's magnetic flux density $\mathbf{B}_{\text{const}}(\mathbf{P})$, gradient force $\mathbf{F}_{\mathrm{B}}$, and current $\mathbf{I}_{\text{const}}$ at point **P**.

$$\begin{bmatrix} \mathbf{B}_{\text{const}}(\mathbf{P}) \\ \mathbf{F}_{\mathrm{B}} \end{bmatrix} = \begin{bmatrix} \tilde{\mathsf{B}}(\mathbf{P}) \\ \mathbf{m}_{\text{rob}}{}^{\mathrm{T}}\widetilde{\mathsf{B}_{\partial x}}(\mathbf{P}) \\ \mathbf{m}_{\text{rob}}{}^{\mathrm{T}}\widetilde{\mathsf{B}_{\partial x}}(\mathbf{P}) \\ \mathbf{m}_{\text{rob}}{}^{\mathrm{T}}\widetilde{\mathsf{B}_{\partial x}}(\mathbf{P}) \end{bmatrix} \mathrm{I} = \mathcal{A}_{\mathrm{B,F}}(\mathbf{m}_{\text{rob}}, \mathbf{P})\mathbf{I}_{\text{const}} \tag{S12}$$

where, as long as the inverse or pseudo-inverse of the contribution matrix $\mathcal{A}_{B,F}(\mathbf{m}_{\text{rob}}, \mathbf{P})$ can be found, it is possible to determine the current required in each coil to produce the desired magnetic field [*55*].

$$\mathbf{I}_{\text{const}} = \mathcal{A}_{\mathrm{B,F}}(\mathbf{m}_{\text{rob}}, \mathbf{P})^{\dagger} \begin{bmatrix} \mathbf{B}_{\text{const}}(\mathbf{P}) \\ \mathbf{F}_{\mathrm{B}} \end{bmatrix} \tag{S13}$$

It should be noted that once the swarm of swimming robots is aggregated at a single point, it can be considered as a single entity for manipulation with multiple degrees of freedom. The dispersal and aggregation of the robot swarm can be achieved by using a custom, completely non-uniform constant magnetic field component with varying directions and magnitudes, but this requires adjusting the excitation current of each Coil 1 according to the task requirements.

Generation of the alternating field by Coil 2

The method to generate the alternating field is similar to that for generating a uniform constant field. According to Eq. (S9), the current $\mathbf{I}_{\text{alter}}$ required to produce a uniform alternating field with a magnetic flux density amplitude $\mathbf{B}_{\text{alter}}$ can be calculated. To maintain a high speed of swimming for the individual robot, in most cases, the direction of the oscillating field's alternating component is perpendicular to that of the constant component.

$$\mathbf{B}_{\text{prop}} \bullet \mathbf{B}_{\text{orien}} = 0 \tag{S14}$$

When the alternating field is rotated around the unit vector $\bar{\mathbf{B}}_{\text{const}}$ of the constant field's flux density, the robot follows the rotation in an axial roll motion to maintain a balance between the magnetic torque and damping force. After a rotation of angle θ_{Roll}, the magnetic flux density of the alternating field can be calculated using Rodrigues' rotation formula.

$$\mathbf{B}_{\text{alter}}' = \mathbf{B}_{\text{alter}}\cos(\theta_{\text{Roll}}) + (\bar{\boldsymbol{B}}_{\text{const}} \times \mathbf{B}_{\text{alter}})\sin(\theta_{\text{Roll}}) + \bar{\mathbf{B}}_{\text{const}}(\bar{\mathbf{B}}_{\text{const}} \cdot \mathbf{B}_{\text{alter}})(1-\cos(\theta_{\text{Roll}})) \tag{S15}$$

S11. Motion control scheme

In addition to maneuvering the millimeter swimming robot via a manual joystick in an open-loop configuration, closed-loop control for trajectory tracking has been implemented, enabling the robot to follow a pre-planned path (Fig. S11a). The robot was precisely controlled to move through a series of target positions derived from segmented paths. The side and top views of its motion are captured by a pair of orthogonally arranged and calibrated CCD cameras. A 3D position observer, developed using LabVIEW-in-built MeanShift algorithm, enhanced positioning accuracy and noise resilience. The robot maintained a

constant velocity and corrected its zenith angle θ_c and azimuth angle φ_c to align its current position (x_m, y_m, z_m) with the predetermined reference points (x_r, y_r, z_r).

$$\begin{aligned} \theta_c &= \arccos\left(\frac{e_z}{\sqrt{\Delta e_x^2 + \Delta e_y^2 + \Delta e_z^2}}\right) \\ \varphi_c &= \arctan\left(\frac{\Delta e_y}{\Delta e_x}\right) \end{aligned} \tag{S16}$$

where $e_x = x_r - x_m$, $e_y = y_r - y_m$, and $e_z = z_r - z_m$ are the errors between the reference point and the current position.

S12. Influence of $\boldsymbol{B}_{\mathrm{alter}}$ on robotic actuation and swarm coordination

We investigated the impact of the amplitude of the alternating component's magnetic flux density (B_{alter}) on the speed and direction of the robot (Fig. S18a). We found that B_{alter} significantly influences the robot's motion speed (Fig. S18b i). Specifically, an increase in B_{alter} leads to an increased tail vibration amplitude A_T (Fig. S18c), which in turn results in greater reaction forces exerted by the fluid for a higher swimming speed of the robot. It is noteworthy that increasing B_{alter} can offset the reduction in the robot's swimming speed caused by the angle between constant and alternating components (θ_B). High B_{alter} ensures that in the collective regulation, each robot maintains sufficient power in the direction controlled by the constant component, enabling it to handle environmental disturbances effectively (Fig. S18b ii).

S13. Influence of $\boldsymbol{B}_{\mathrm{const}} : \boldsymbol{B}_{\mathrm{alter}}$ on robotic actuation and swarm coordination

We have analyzed the impact of the ratio of magnetic flux density's norm between constant and alternating components ($B_{\mathrm{const}} : B_{\mathrm{alter}}$) on the speed and direction of the robot (Fig. S19a). It is observed that $B_{\mathrm{const}} : B_{\mathrm{alter}}$ has little effect on speed and direction when the magnetic flux density of the constant component is lower than that of the alternating component (Fig. S19b i and ii). Conversely, if B_{const} exceeds B_{alter}, for instance at a 2:1 ratio, a noticeable decrease in speed occurs, along with unpredictable direction. The robot's midlines over a singular vibrational period have been extracted. Subsequent alignment of these midlines' tips with the vertical axis is employed to quantify the tail amplitude A_T (Fig. S19c i). Conversely, alignment with the coordinate origin has been utilized for the assessment of the angular displacement of the head θ_H (Fig. S19c

ii). With a magnetic flux density ratio below 1, the robot's head exhibits a smaller swinging angle θ_H compared to the composite oscillating magnetic field. This phenomenon occurs because the head, moving rapidly in an attempt to follow the oscillating field with the larger swinging angle, encounters increased damping forces. Under a limited actuation field, this leads to the desynchronization of the robot's head with the magnetic field, similar to the rotating magnetic robots above the step-out frequency [57]. In such scenarios, when the swinging angle of the oscillating field changes, both the head's swinging angle θ_H and the tail amplitude A_T change by a small amount, leading to insignificant changes in speed and motion direction. In contrast, when the ratio exceeds 1, the robot's head synchronizes with the oscillating magnetic field, maintaining consistent swinging angles. As this ratio $B_{const} : B_{alter}$ increases, the composite field's swinging angle decreases, leading to a corresponding reduction in the robot's head swinging angle θ_H and tail amplitude A_T, which slows down the speed and makes the direction unpredictable. However, the constant component $\mathbf{B}_{const}$ must not be too small, otherwise the robot will not be able to swim (Fig. S20). Therefore, it is essential to regulate the magnetic flux density ratio between constant and alternating components $B_{const} : B_{alter}$ to ensure that the constant component does not interfere with propulsion.

S14. Reduction and testing of adhesion strength

The impact of fetal bovine serum immersion on the adhesive properties of silicone surfaces was investigated. The robots were immersed in fetal bovine serum for 2 hours. Adhesion tests were conducted using a customized displacement-force test station equipped with a force sensor (Futek 538493). A pair of silicone samples (26 mm in diameter), treated in the same manner, were individually affixed to the top and bottom glass substrates using adhesive. A pre-load of 12.5 N was applied to ensure adequate contact. The silicone contact surfaces were slowly separated at a rate of 0.1 mm s^{-1}, and the corresponding force-displacement curves were recorded. The adhesion strength was calculated by dividing the maximum force measured by the contact area [58]. As evidenced by the results presented in Fig. 5c, it was observed that the adhesion strength of silicone significantly decreases following immersion in fetal bovine serum.

S15. Determination of connectivity status

As illustrated in Fig. 5a, robotic connection generally involves side contact, with the head of the rear robot aligning at the junction of the head and tail of the front robot, firmly connected under the action of surface adhesion force (F_{adh}). Disregarding magnetic forces, the connected robots are also subjected to fluid resistance (F_r) during oscillation, which may cause separation. In the 2D model, the adhesion force is determined by the material's adhesion strength (P_{adh}) and the contact length (l_{adh}). Ignoring viscous resistance, the dominant component of fluid resistance is pressure drag, influenced by the robot's rotational speed (ω_s), oncoming length (l_r), drag coefficient (C_d), and fluid density (ρ_f). The adhesion force and fluid resistance of connected robots are related as follows:

$$\int_0^{l_{adh}} P_{adh} l \cdot \mathrm{d}l = \int_{l_{adh}}^{l_{adh}+l_r} 0.5 C_d \rho_f (\omega_s l_{rot})^2 l \cdot \mathrm{d}l \qquad \text{(S17)}$$

where, l_{adh} represents the effective length over which adhesion force acts. Due to the robot's low flexural strength, the adhesive force that counters fluid resistance is concentrated within a limited area rather than across the entire contact area, thus being set to 10 % of the robot's tail length. l_r denotes the length of the oncoming flow region, which for simplicity, is equal to the length of the robot's head. Given the robot's thin-film shape, the drag coefficient C_d is set to 1. The centroid of the connected robots is considered as the center of rotation, l_{rot} is the distance to the centroid. Using Eq. (S17), the relationship between the robots' connectivity can be determined based on the rotational angle ω_s and adhesion strength P_{adh}. Fig. 5b shows that in the lower right corner, due to high rotational speeds, adhesive force is insufficient to counteract fluid resistance, leading to disconnection; in the upper left corner, higher adhesion strengths enable robots to maintain connection.

We extracted the midlines of firmly connected robots under oscillating magnetic fields with alternating components of 3 mT and 9 mT at 31.1 Hz (Fig. S21a), and calculated their rotational speeds ω_s from the envelope of the midline, allowed us to determine, based on the model, that the robots would disconnect at 9 mT. The experimental results (Fig. S21b) confirmed that surface-treated robots remained connected under weak alternating fields (3 mT) but disconnected when B_{alter} increased to 9 mT, validating our model results (Fig. 5b).

S16. Simulation of magnetic force between two robots

We utilized commercial finite element software (COMSOL) for the 3D magnetic force between two robots. Detailed simulation parameters are provided in Table S4. Two robots with consistent heights (z) were positioned at distances $Dist_x$ and $Dist_y$ in the x and y directions, respectively. Using the Force Calculation interface, the magnetic force $F_{B\text{-}y}$ between the robots at different distances was calculated (Fig. 5e). When $F_{B\text{-}y}$ is positive, the two robots attract each other. It is evident that as $Dist_y$ decreases, the magnetic force increases significantly. Additionally, it can be observed that the two robots repel each other when $Dist_y$ is close to 0 mm, while the magnetic attraction is maximized when $Dist_y$ is around -1.3 mm. This indicates that the two robots tend to connect at this position ($Dist_y$ = -1.3 mm), which is consistent with the experimental results.

S17. Reciprocating motion model of the swimming robot

As shown in Fig. 7c, the robot's reciprocating motion on the interface is primarily influenced by propelling force (F_p), gravity (G), buoyancy (F_b), fluid resistance (F_r), supporting force (F_s), and magnetic torque (T_m). To streamline the analysis, it is assumed that the robot has no deformation and its posture at position x aligns with the symmetry axis of the midline envelope region at that position ($\beta_e = \beta_r$). The angle (β_r) between the symmetry axis of the midline envelope region and the interface determines the robot's turning point. At the region's boundary, the angle β_r reached 90°, which indicated that the robot switched motion direction (Fig. S24e). Using static analysis, we can approximately calculate β_e at different x, thereby determining the length (L_r) of the reciprocating motion region. At position x, the torques acting on the robot are in equilibrium, which can be expressed as follows:

$$T_m + F_b \cdot l_f \cos(\beta_e) = F_r \cdot l_f \sin(\beta_e) + G \cdot l_g \cos(\beta_e) \quad \text{(S18)}$$

where, l_g and l_f represent the distances from the robot's center of gravity and center of form to the head, respectively; β_e denotes the angle between the robot and the interface. The magnetic torque T_m exerted on the robot with a magnetic moment $\mathbf{m}_{rob}$ can be calculated using Eq. (S6). The flux density's norm (B_{const}) of the constant component at position x is determined by a calibrated dipole model using Eq. (S5). The fluidic

resistance F_r encountered by the robot during its reciprocating motion predominantly consists of viscous drag and pressure drag. For model simplification, the influence of viscous drag is neglected, thereby simplifying the fluidic resistance to pressure drag [51]:

$$F_r = 0.5 \cdot C_d \cdot \rho_f \cdot S_{rob} \cdot v_h^2 \tag{S19}$$

where, C_d represents the drag coefficient, which is contingent upon the shape of the robot, its surface roughness, and the Reynolds number of the fluid; ρ_f denotes the density of fluid; $S_{rob} = h_{rob} \cdot l_{rob} \cdot \sin(\beta_e)$ corresponds to the frontal area of the microbot, where h_{rob} and l_{rob} represent the length and height of the robot, respectively; $v_h = v \cdot \cos(\beta_e)$ signifies the component of the robot's swimming velocity v that is parallel to the interface. The swimming velocity v is dictated by the parameters of the magnetic field. Calculations were conducted for the constant and alternating components within the robot's reciprocating motion region (Fig. S25d). It can be seen that the maximum flux density of the constant component within this region was 4 mT (U_{const} = 20 V), while the flux density amplitude's norm (B_{alter}) of the uniform alternating component was above 4 mT. Given that the ratio of the magnetic flux densities's norm of the constant to alternating field components B_{const} : B_{alter} did not exceed 1, the influence of this ratio on v was deemed negligible (Fig. S25a). Furthermore, the calculated maximum deviation of the angle θ_B between the constant and alternating components from 90° was approximately 15°. Fig. S25b illustrated that minor variations in the angle had a minimal impact on v, thus the effect of the angle can also be disregarded. As depicted in Fig. S25c, v was directly proportional to the flux density amplitude's norm (B_{alter}) of the alternating component. By integrating Eq. (S6) and Eq. (S19) into Eq. (S18), the angle β_e at position x can be determined. At B_{alter} = 10 mT and U_{const} = 8 V, the relationship between position x and angle β_e was calculated (Fig. S25e), similar to the experimental results. By changing U_{const} and B_{alter}, the corresponding length (L_r) of the reciprocating motion region can be calculated (Fig. S25f), which is also close to the experimental results. The slight difference from the experimental results may be due to the neglect of viscous drag. As shown in Fig. S25f enhancing B_{alter} increases L_r. This is because increasing B_{alter} raises the swimming speed, leading to an increase in fluid resistance F_r. The robot only reverses direction (β_r = 90°) when it moves far enough to

receive sufficient torque T_m to counteract the torque induced by F_r. Additionally, decreasing U_{const} also increases L_r. This occurs because reducing U_{const} decreases the T_m applied to the robot, requiring it to move further to gain enough T_m to overcome the torque due to F_r, thereby facilitating the turnback.

Supplementary Note 2 Supplementary Tables

Table S1 Summary of variables on the magnetic field and the magnetic actuation system

Variable	Explanation	Unit
$\mathbf{B}_{\mathrm{const}}$	Magnetic flux density of a constant field	T
B_{const}	The norm of the constant field's magnetic flux density	T
$\mathbf{B}_{\mathrm{alter}}$	Magnetic flux density amplitude of an alternating field	T
B_{alter}	The norm of the alternating field's magnetic flux density amplitude	T
f	The frequency of the actuation field	Hz
ω	The angular frequency of the actuation field	rad
θ_{B}	The angle between the constant and alternating fields	degree
U_{const}	The excitation voltage on Coil1 for the constant field	V
U_{alter}	The excitation voltage on Coil2 for the alternating field	V
$\mathbf{I}_{\mathrm{const}}$	Current of the 8 Coil 1s for constant magnetic field component	A
$\mathbf{I}_{\mathrm{alter}}$	Current of the 8 Coil 2s for alternating magnetic field component	A
$\mathbf{m}_{\mathrm{d}}$	Magnetic moment of the dipole	A m^2
$\mathbf{P}_{\mathrm{m}}$	Location of the dipole	m
$\tilde{\mathbf{B}}_{\mathrm{orien}-i}(\mathbf{P})$	The magnetic flux density of the constant field at the target point **P**, generated by the i-st Coil 1	T
θ_{Roll}	Rotation angle of alternating field around constant field	degree

Table S2 Summary of variables of the individual robot

Variable	Explanation	Unit
$\mathbf{m}_{\mathrm{rob}}$	The robot's magnetic moment	A m^2
$\mathbf{T}_{\mathrm{m}}$	The magnetic torque	N m
T_{m}	The norm of magnetic torque	N m
$\mathbf{F}_{\mathrm{B}}$	The magnetic force	N
F_{B}	The norm of magnetic force	N
$\mathbf{v}$	The robot speed	m s^{-1}
v	The norm of the robot speed	m s^{-1}
A_{H}	Vibration amplitude of robot head	m
A_{T}	Vibration amplitude of robot tail	m
θ_{H}	The swinging angle of the robot's head	degree
$\theta_{\mathrm{v\text{-}B}}$	The angle between the constant field and the robot velocity vector	degree
β_{m}	The angle between the symmetry axis of the midlines' envelope region and the direction of constant fields	degree
θ_{s}	Angle of overall swing	degree
ω_{s}	rotational speed	rad s^{-1}

Table S3 Summary of variables of the robot swarm

Variable	Explanation	Unit
L_r	Length of the reciprocating motion region	m
β_e	The angle between the robot and the interface	degree
β_r	The angle between the symmetry axis of the midline envelope region and the interface	degree
β_s	The tilt angle of the interface	degree
F_r	The norm of fluid resistance	N
P_{adh}	Adhesion strength	Pa
F_{imp}	Fluid impact pressure	N
v_f	flow velocity	m s^{-1}

Table S4 Summary of simulation parameters

**Fluid**		
μ_f	0.001 [Pa s]	Fluid dynamic viscosity
ρ_f	1000 [kg m^{-3}]	Fluid density
K_f	2.2×10^9 [Pa]	Fluid bulk modulus
**Robot**		
h_{rob}	0.5 [mm]	Robot height
l_{rob}	2 [mm]	Robot length
t_{rob}	30 [μm]	Robot thickness
R_{HT}	13 : 7	Head-to-tail ratio
ρ_H	1250 [kg m^{-3}]	Robot head density
ρ_T	1070 [kg m^{-3}]	Robot tail density
E_H	147.33 [kPa]	Robot head elasticity modulus
E_T	78.95 [kPa]	Robot tail elasticity modulus
N_H and N_T	0.45	Head and Tail Poisson's ratio
M_H	17 [kA m^{-1}]	Robot head Magnetization strength
$Dist_{x-in}$	1.3 [mm]	Initial relative distance in the x
$Dist_{y-in}$	1 [mm]	Initial relative distance in the y
**Transversal displacement**		
γ	2094.40 [rad m^{-1}]	Wave number
ω	195.41 [rad s^{-1}]	Robot frequency
t_a	0.2 [s]	Activation characteristic time
k_1	0.540 (0.252)	Envelope amplitude coefficients at B_{dyn}=9mT (3mT)
k_2	-0.644 (-0.291)	
k_3	0.284 (0.124)	

The elasticity modulus of the robot head (hard magnetic elastomer, 0.2 mass ratio of NdFeB powder to 00-30) was measured by Atomic Force Microscopy (AFM). The robot tail's elasticity modulus (pure 0030) was provided by the manufacturer (smooth-on.com). The magnetic properties of the NdFeB powder (MQFPTM-15-7-20065-089), with the magnetization curve provided by the manufacturer (mqitechnology.com). The robot head magnetization strength can be calculated based on the ratio of magnetic powder and 0030

Supplementary Note 3 Supplementary Figures

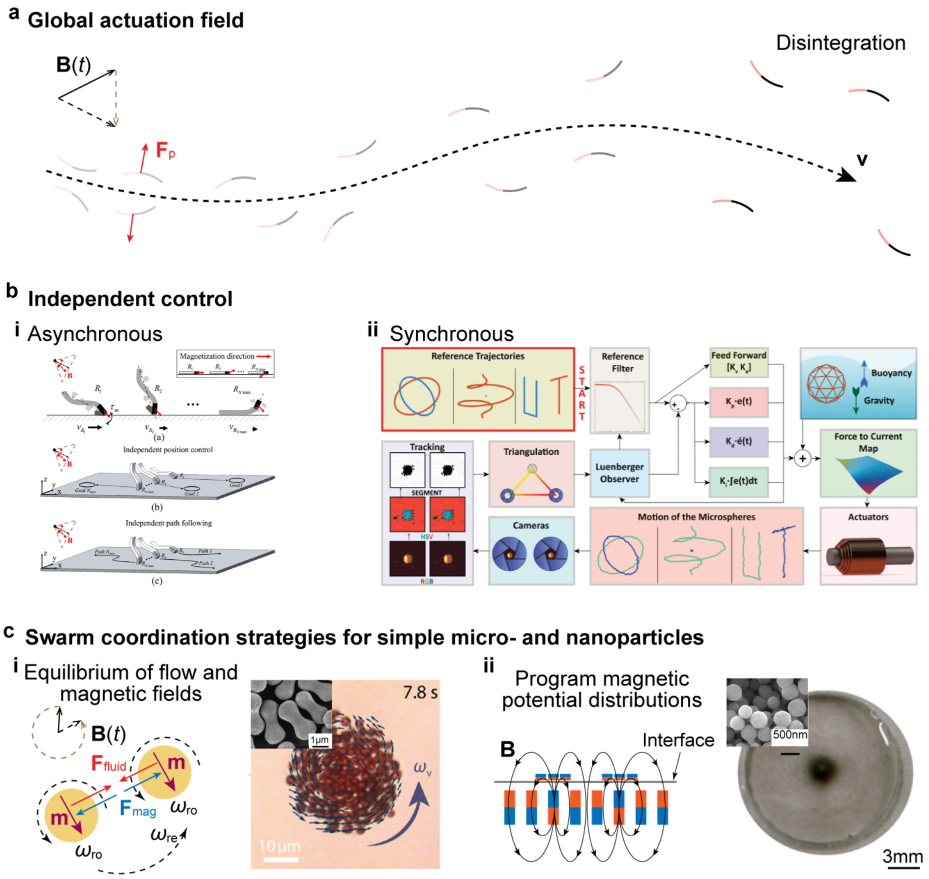

Fig. S1. Swarm coordination strategies for magnetic soft robot. (a) Under global actuation field, the swarm of miniature magnetic soft robots gradually disintegrates due to lateral pressure and individual differences. (b) (i) Asynchronous [9] and synchronous [12] independent control of a small number of individuals. (c) Swarm coordination strategies for simple micro- and nanoparticles. (i) Swarm formation by equilibrium of flow and magnetic fields [13]. (ii) Swarm formation by programming magnetic potential distributions [59].

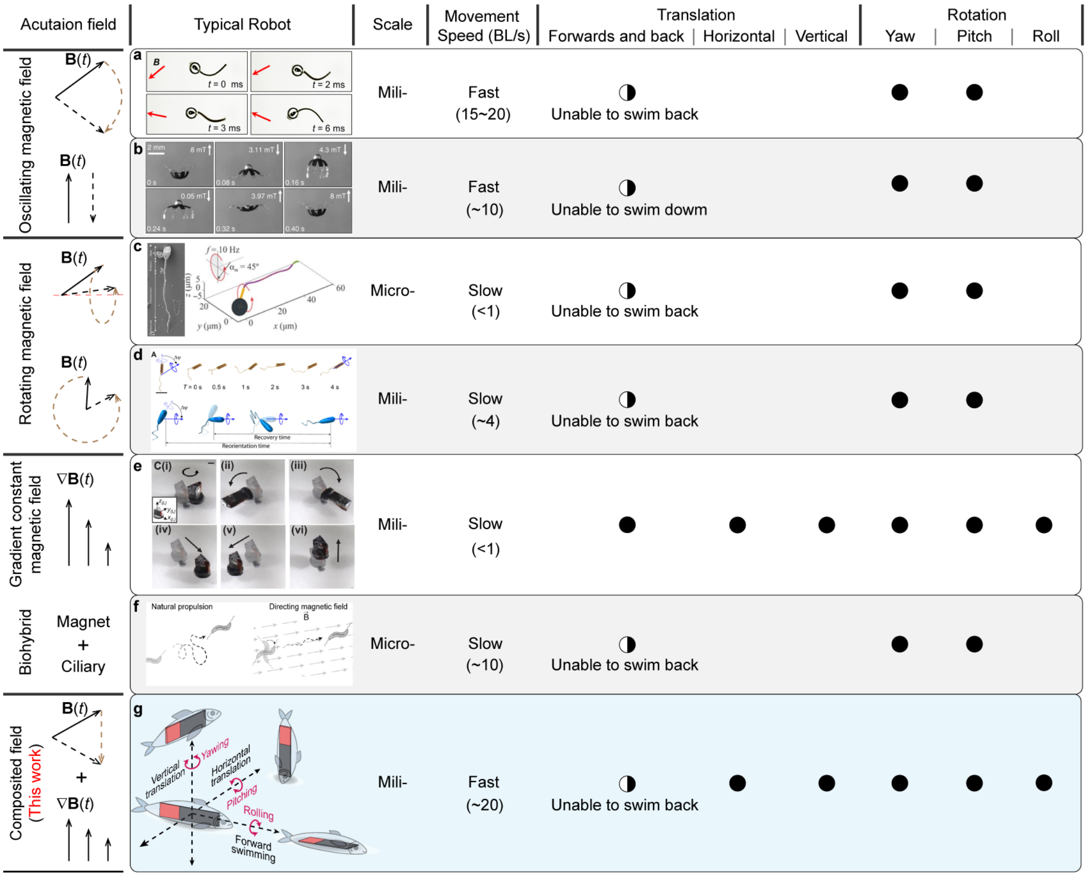

Fig. S2. Comparison with existing typical individual swimming robots. (a) The miniature fish-like robot under the directional oscillating magnetic field [2]. (b) The miniature jellyfish-like robot under the unidirectional oscillating magnetic field [3]. (c) Sperm-templated soft magnetic microrobots under the directional rotating magnetic field [19]. (d) Bacteria-inspired helical-motion robot under the unidirectional rotating magnetic field [21]. (e) 6-DOF motion robot under the gradient constant magnetic field [23]. (f) Soft biohybrid microrobots with magnetic field for direction and ciliary for propulsion [17]. (g) Our robot is under a composed field, including an oscillating magnetic field and a gradient constant magnetic field.

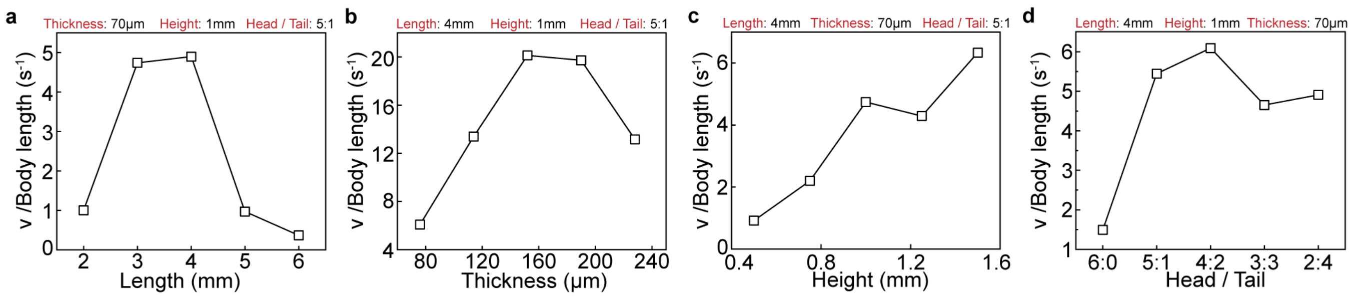

Fig. S3. Dependency of relative velocity on physical dimensions. (a) Length, (b) Thickness, (c) Height, and (d) Head-to-tail ratio vs. relative velocity. at B_{const} = 1 mT, B_{alter} = 5 mT, and f = 31.1 Hz.

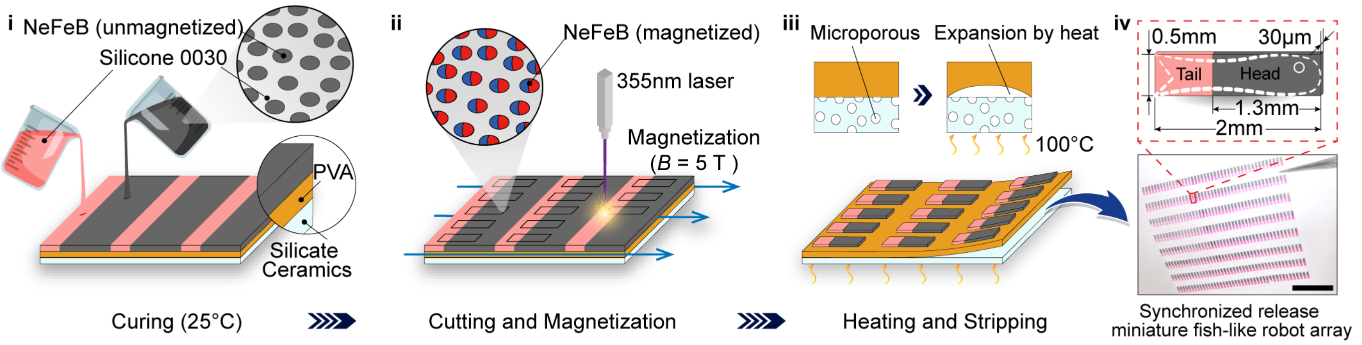

Fig. S4. Mass fabrication of miniature fish-like magnetic soft robots. (i) Pure silicone 0030 (red), and 0030 mixed with unmagnetized NdFeB (black), are cured at room temperature on silicate ceramics coated with PVA. (ii) A 355 nm laser is utilized to precisely cut the robot array and a 5 T pulsed magnetic field is applied to magnetize the NdFeB particles. (iii) Heating induces the expansion of gases within the microporous ceramic, which facilitates the detachment of the PVA film with the robots from the ceramic substrate. (iv) The synchronized release miniature fish-like robot array is obtained.

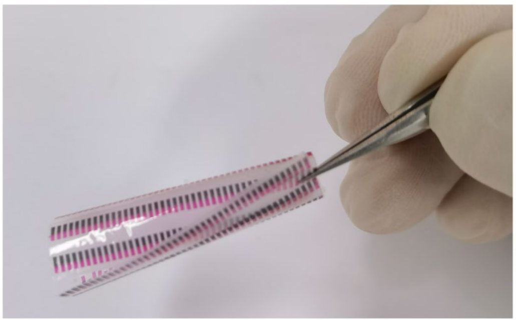

Fig. S5. A rolled-up SyncRelease miniature fish-like robot array. The soft PVA layer allows the SyncRelease miniature fish-like robot array to be rolled up and then transported to the designated location through a catheter.

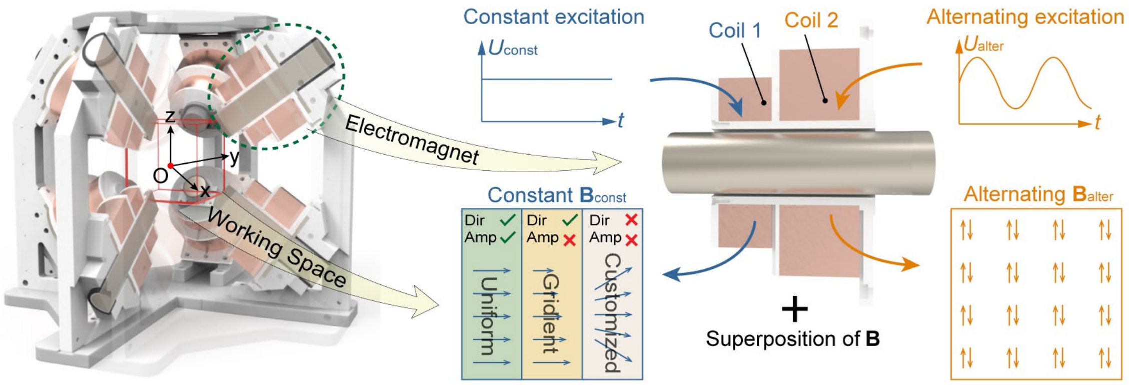

Fig. S6. Generation of composite magnetic fields. Diagram of an electromagnetic apparatus producing composite magnetic fields. It features an eight-electromagnet setup. Each electromagnet has two independently excited coils for generating constant and alternating magnetic fields. Superimposing the magnetic fields generated by eight electromagnets enables the simultaneous creation of a uniform alternating field and three types of constant fields (uniform, gradient, and customized) in the workspace.

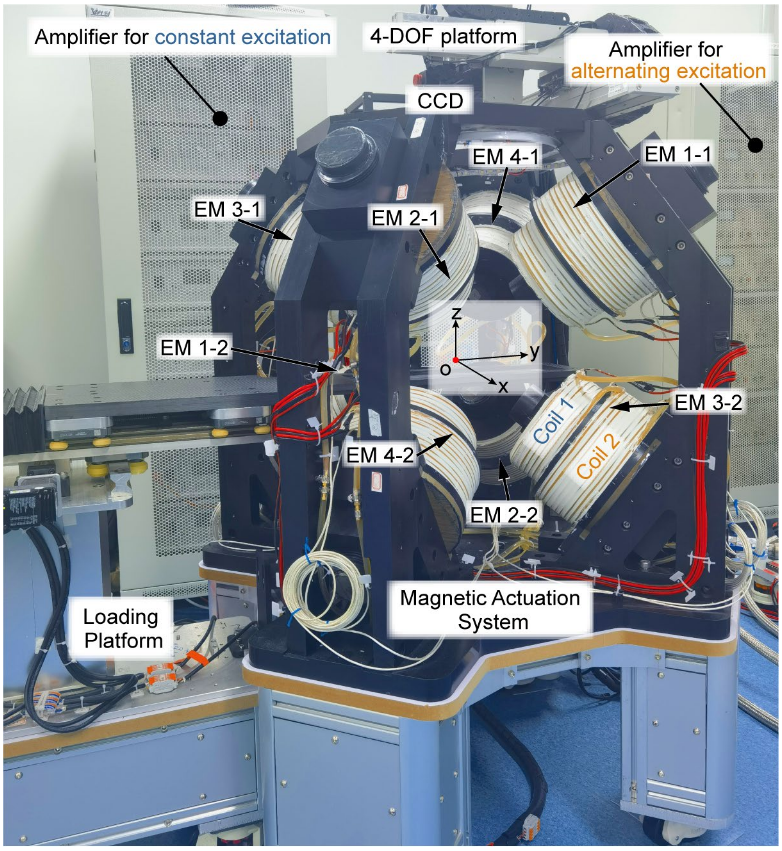

Fig. S7. Physical setup of the experimental apparatus for magnetic actuation. This photograph showcases the actual experimental setup comprising the magnetic actuation system, loading platform, amplifiers for constant and alternating excitation, 4-DOF platform, and a CCD camera. The magnetic actuation system consists of eight electromagnets arranged in pairs across four groups. For instance, in group 1, the electromagnet mounted above is labeled "EM 1-1," while its counterpart fixed to the base is denoted as "EM 1-2." Each electromagnet is composed of a coil and core, with Coil 1 and Coil 2 designed to generate constant and alternating fields, respectively.

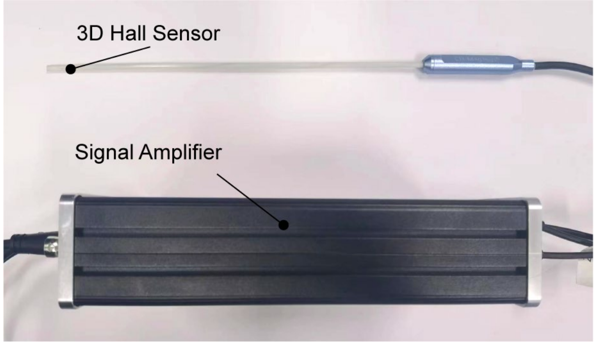

Fig. S8. 3D Hall sensor. The signal from the sensor is amplified and captured by the host computer. The Hall sensor probe was fixed on a 4-DOF platform and traversed the workplace of the magnetic actuation system.

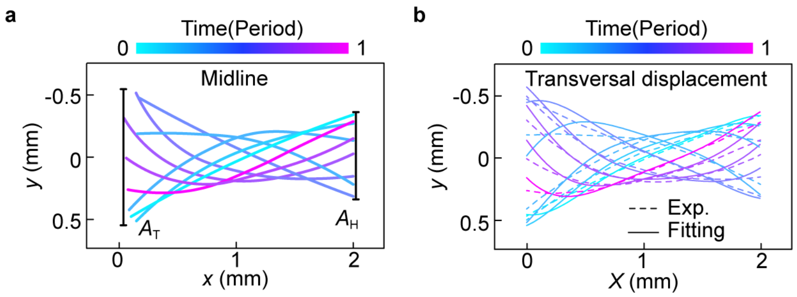

Fig. S9. Motion analysis of the individual robot within one motion cycle. (a) The midlines were extracted and then aligned such that snout tips remained on a horizontal line. The head's vibration amplitude (A_H) was less than the tail's (A_T), resulting in a stronger propulsive force from the tail, enabling forward motion. (b) The transversal displacement of the robot at each position. The motion model can be fitted by transverse displacements.

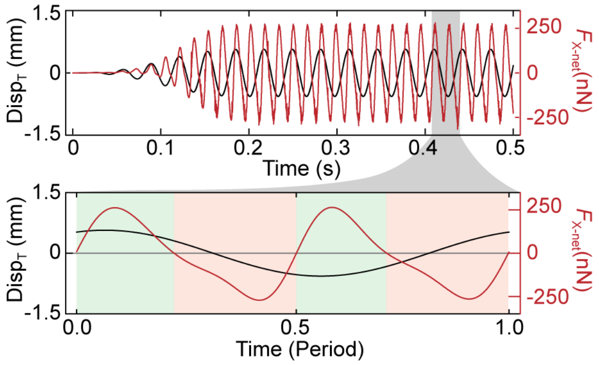

Fig. S10. Analysis of simulation results. The net force along the forward direction ($F_{\text{x-net}}$) and the displacement of the tail (Disp_T) were recorded over a total simulation time of 0.5 s. One complete flap of the tail produces two forward thrusts. When the robot is in steady motion, the integral of the net force in one motion cycle is zero.

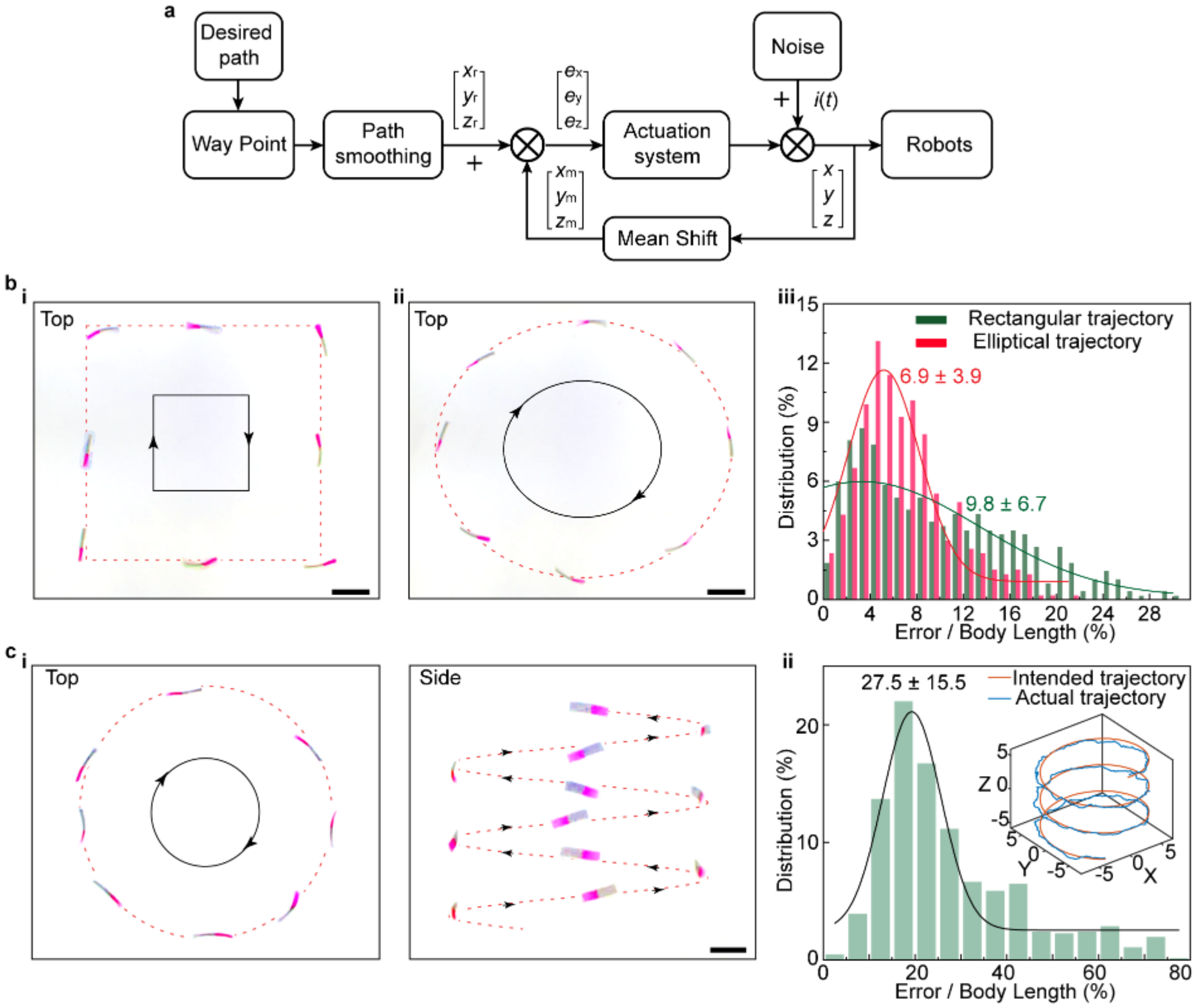

Fig. S11. Trajectory tracking of the individual swimming robot. (a) Schematic diagram of the control system for locomotion. A closed-loop controller with integration of image processing, path planning, and motion control algorithms, was designed for trajectory tracking. Two calibrated CCDs were placed orthogonally for the spatial location of the robot. Here, (x, y, z), (x_m, y_m, z_m), and (x_r, y_r, z_r) represent actual, measured, and reference locations of the robot. (e_x, e_y, e_z) represent location errors and $i(t)$ is the noise from the surroundings. (b) 2D trajectory tracking. Images synthesized by movie screenshots illustrate the locomotion results of the robot in tracking planned trajectories of (i) a rectangle and (ii) an ellipse. Distribution histograms showing the tracking error (with SD) of 9.8 ± 6.7 %, and 6.9 ± 3.9 % for rectangular and elliptical trajectories. (b) 3D trajectory tracking. (i) Images synthesized by movie screenshots of a robot tracking a planned helical trajectory. (ii) The distribution histogram shows a tracking error (with SD) of 27.5 ± 15.5 %. All scale bars, 2 mm.

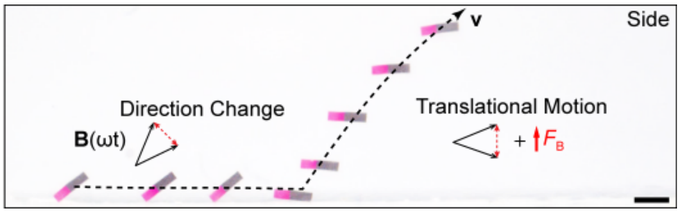

Fig. S12. Translational motion of the individual swimming robot. The vertical translation empowered the robot to overcome gravity and ascend when power was insufficient for propulsion. Scale bar, 2 mm.

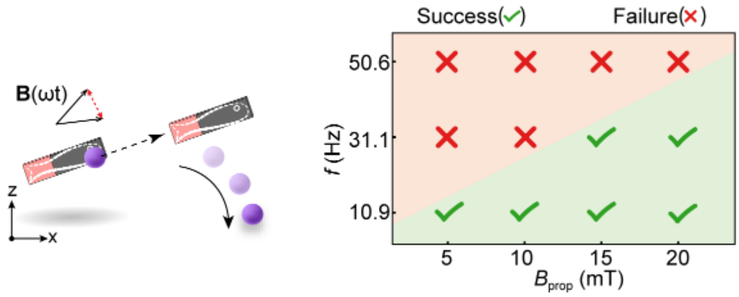

Fig. S13. The ability of the robot to shake off objects. As the frequency decreases and the magnetic flux density increases, the robot's vibration amplitude grows, facilitating easier detachment of the object.

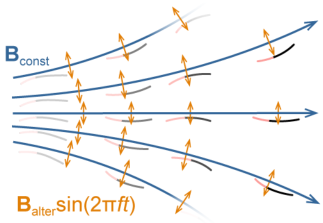

Fig. S14. Directional coordination of robotic swarms based on everywhere orthogonal constant and alternating magnetic field components

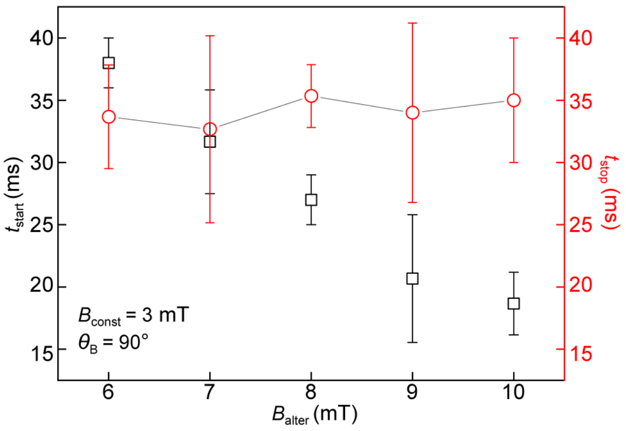

Fig. S15. The start time t_{start} and stop time t_{stop} of the robot.

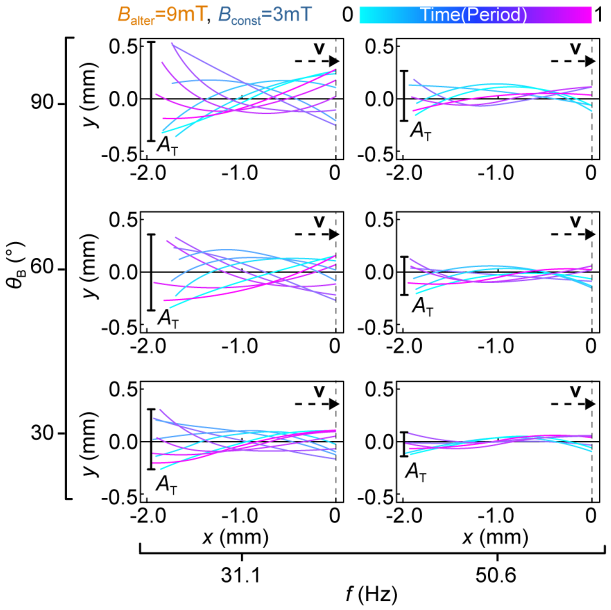

Fig. S16. The midline of the robot at different combinations of f and θ_B. The snout tips of all midlines were aligned to a vertical line, facilitating the measurement of the tail amplitude A_T. The tail amplitude A_T decreases with an increase in actuation frequency f or a reduction in the angle θ_B between the constant and alternating components.

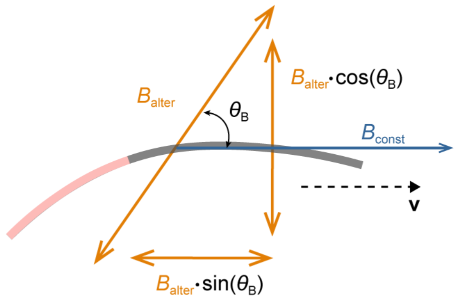

Fig. S17. Schematic diagram of alternating magnetic field component decomposition. When vibrating, the swimming direction of the robot is aligned with the constant component $\mathbf{B}_{const}$. The alternating component $\mathbf{B}_{alter}\sin(\omega t)$ can be further decomposed into components perpendicular and parallel to the robot.

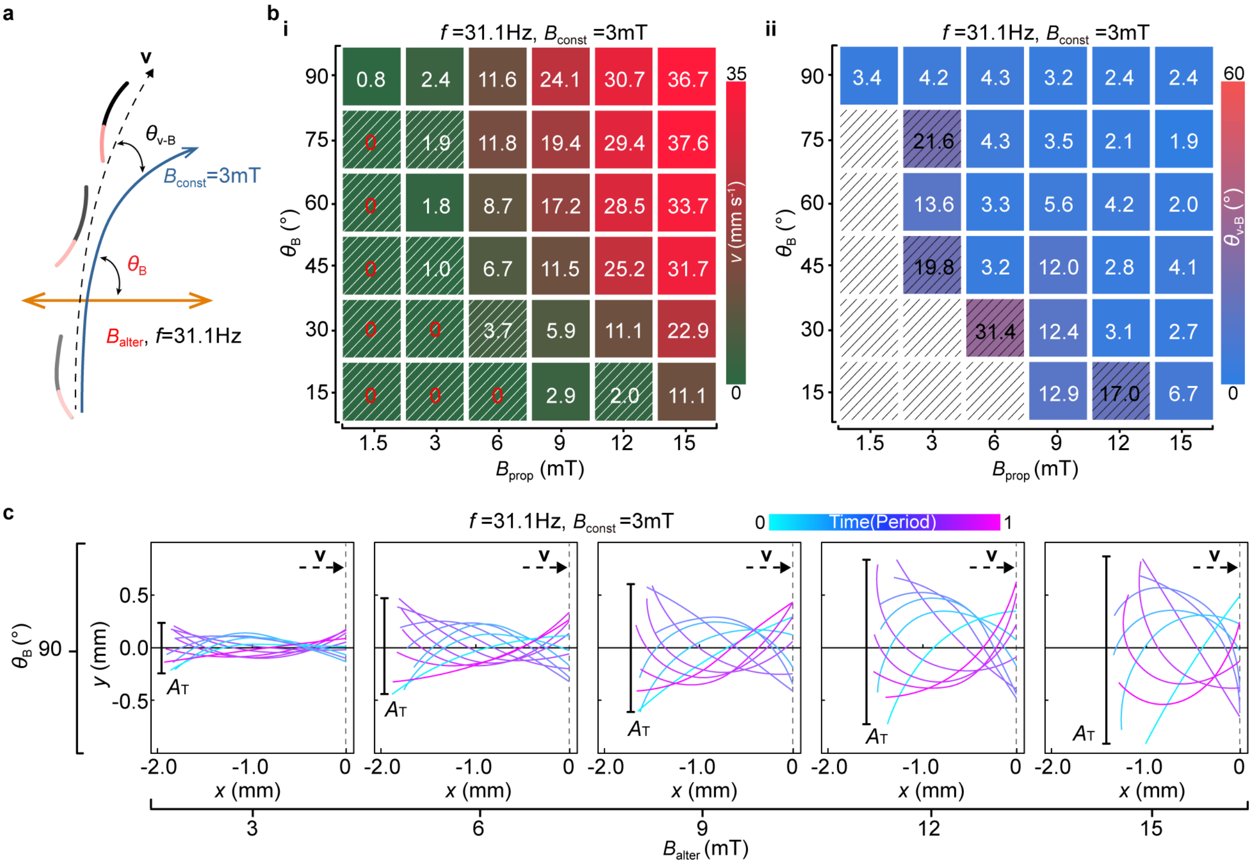

Fig. S18. Miniature fish-like magnetic soft robot's speed and direction at various B_{alter}**.** (a) Schematic of the robot's motion driven by a composite magnetic field consisting of constant and alternating components. (b) Influence of the alternating magnetic field component's magnetic flux density B_{alter} on (i) robot speed v and (ii) the angle between the speed and the constant component $\theta_{\text{v-B}}$. Impractical combinations ($v = 0$ or $\theta_{\text{v-B}} > 15°$) are indicated by slashes. (c) The midline of the robot during a motion cycle at different B_{alter}, with the snout tips of all midlines aligned to a vertical line. The amplitude of the tail A_T increased with an increase in the alternating component's magnetic flux density B_{alter}.

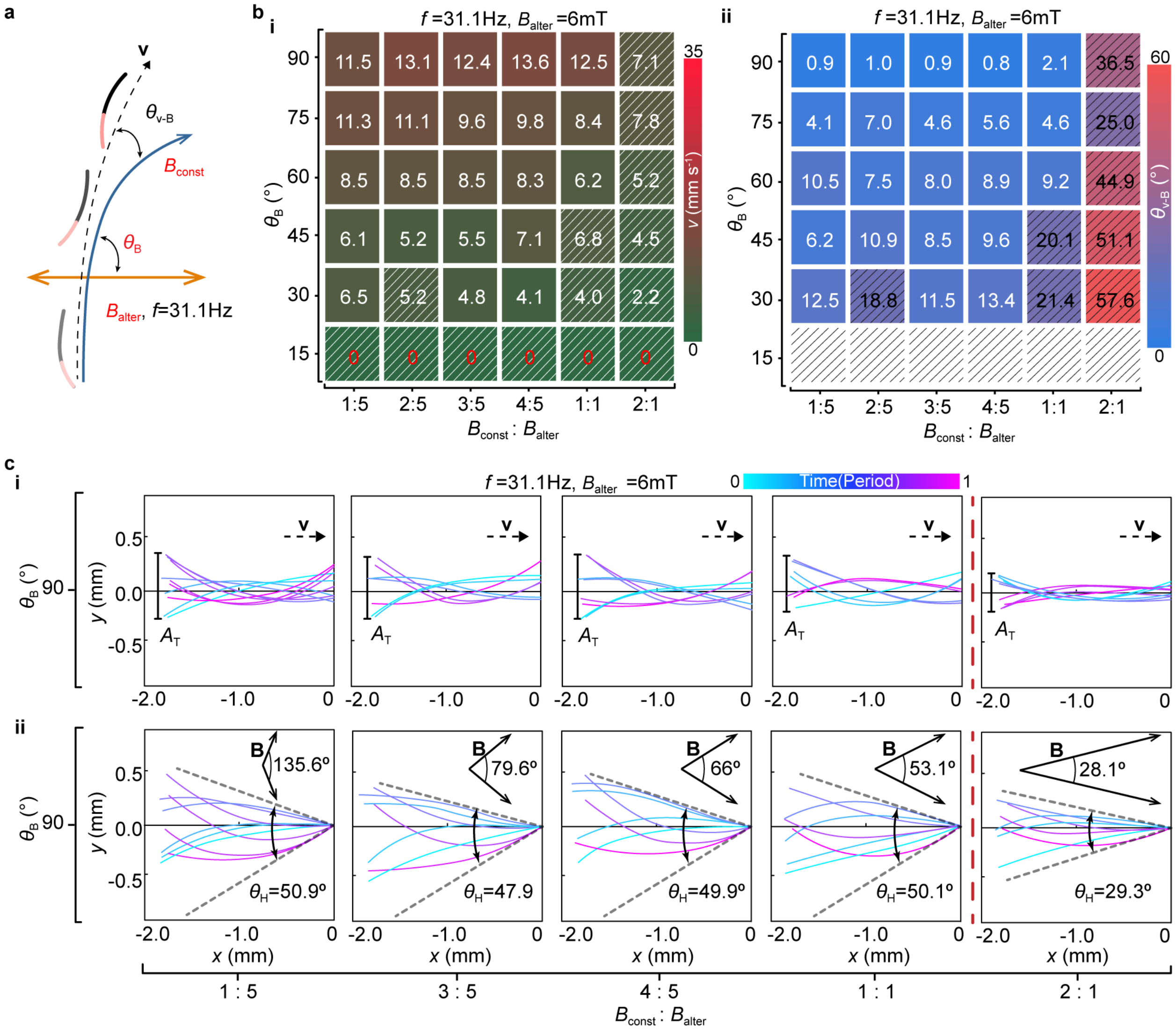

Fig. S19. Miniature fish-like magnetic soft robot's speed and direction at various $B_{const} : B_{alter}$. (a) Schematic of the robot's motion driven by a composite magnetic field consisting of constant and alternating components. (b) Influence of the ratio of flux densities of constant and alternating fields $B_{const} : B_{alter}$ on (i) robot speed v and (ii) the angle between the speed and the constant component $\theta_{v\text{-}B}$. Impractical combinations ($v = 0$ or $\theta_{v\text{-}B} > 15°$) are indicated by slashes. (c) The midline of the robot during a vibration cycle at different $B_{const} : B_{alter}$. (i) Alignment of all midlines' snout tips to a perpendicular axis enabled precise quantification of tail amplitude A_T. (ii) The snout tips of all midlines were aligned together to the coordinate origin, forming the swinging angle θ_H. No significant change in tail amplitude A_T and swinging angle θ_H when $B_{const} : B_{alter}$ was less than 1:1. While the tail amplitude and swinging angle decreased with $B_{const} : B_{alter}$ greater than 1:1.

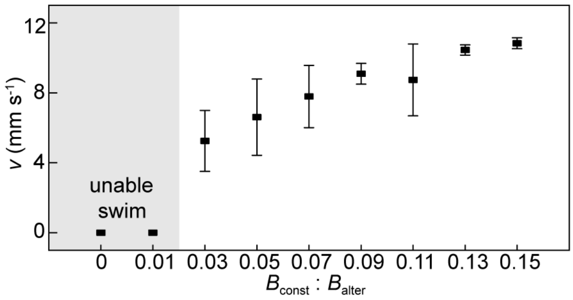

Fig. S20. Effect of smaller B_{const} : B_{alter} on swimming speeds.

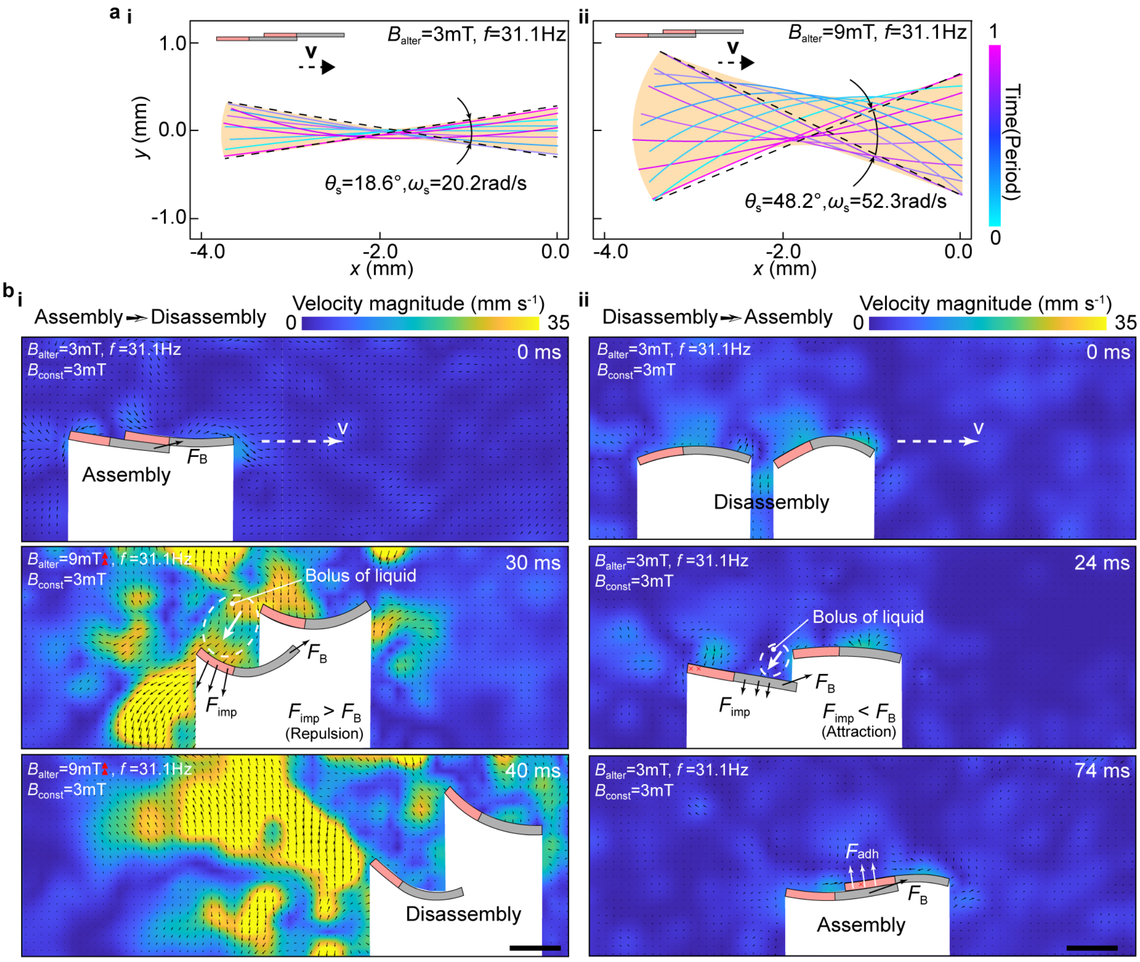

Fig. S21. Two swimming robots' assembly and disassembly. (a) Midline trajectories of two securely connected robots within a single vibration cycle, under alternating components of (i) 3 mT and (ii) 9 mT at 31.1 Hz, achieving head alignment. The envelope area of these midlines, marked in yellow, enabled the determination of the robots' rotational angles (θ_s) throughout a motion cycle, allowing for the calculation of their rotational speeds (ω_s). The rotational speed increases with the rise of the alternating component. (b) PIV captured the velocity vector fields (black arrows) around two robots during (i) assembly or (ii) disassembly realized at B_{alter} = 3 or 9 mT, respectively. The velocity magnitude (color map) was also calculated. Lateral fluid impact pressure on the trailing robot from the leading robot's rearward flapping causes repulsion between the robots, whereas their magnetic force facilitates mutual attraction. The relationship between impact pressure (F_{imp}) and magnetic force (F_B) magnitude dictates whether two separated robots are attracted toward each other or repelled away. The larger the alternating components, the faster the fluid flow, leading to dominant impact pressure and resulting in the two robots repelling each other. All scale bars, 1 mm.

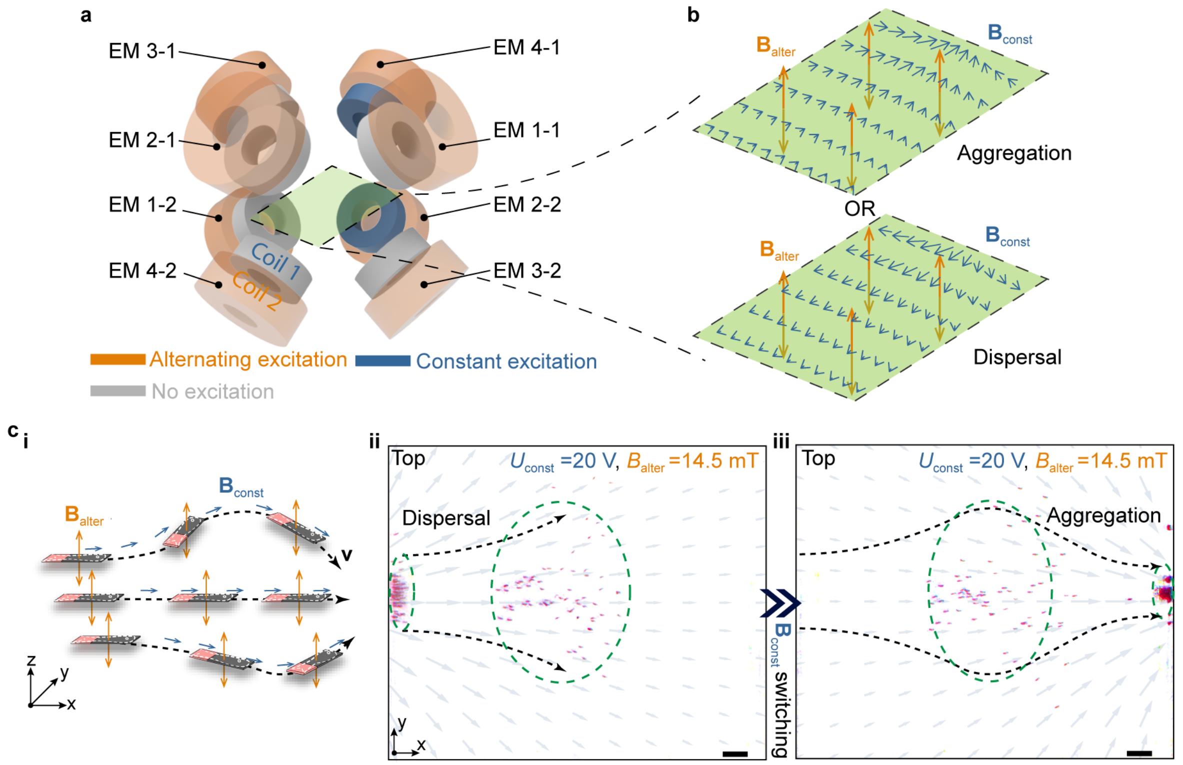

Fig. S22. The aggregation and dispersal of the robot swarm. (a) The activation scheme within an electromagnetic array to produce a composite magnetic field. The cores are not shown for clarity. All Coil 2s were dynamically excited to generate a nearly uniform alternating magnetic field component (B_{alter}). Pairs of adjacent Coil 1s (e.g., EM 4-1 Coil 1 and EM 2-2 Coil 1) received stable excitation (U_{const}) to create a completely non-uniform constant magnetic field component (B_{const}). (b) Alternating the direction of the constant current excitation allows for the manipulation of the non-uniform field's orientation, enabling the switch between aggregated and dispersed states of the robot swarm. (c) Dispersal and aggregation. (i) The schematic of the robot swarm's dispersal or aggregation along a diverging or converging magnetic field. (ii) and (iii) Experimental demonstration of the robot swarm's dispersal followed by aggregation at a target location through the switching of the constant component. Scale bar, 10 mm.

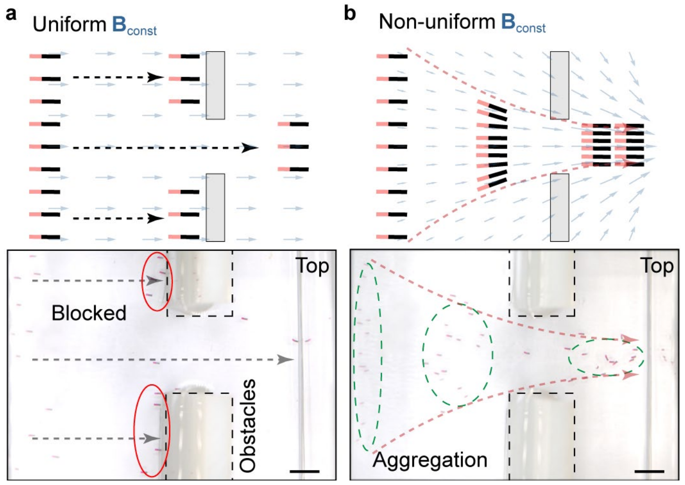

Fig. S23. Navigation through the obstacle. In contrast to (a) the partially blocked robot swarm under the uniform constant component $\mathbf{B}_{\mathrm{const}}$, the robot swarm aggregated under the non-uniform constant component $\mathbf{B}_{\mathrm{const}}$ can pass through the unique channel. Scale bar, 10 mm.

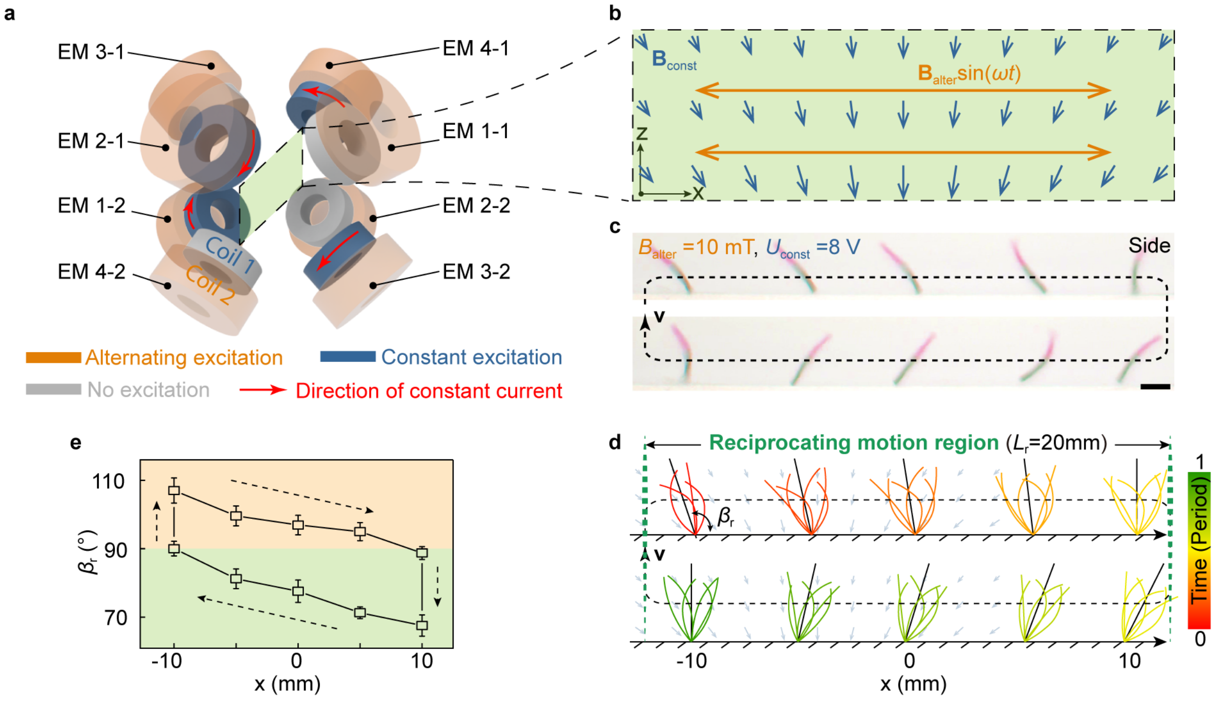

Fig. S24. The morphology regulation and locomotion of the robot swarm. (a) The activation scheme within an electromagnetic array for the generation of a composite magnetic field. The cores are not shown for clarity. All Coil 2s were dynamically excited to produce a nearly uniform alternating component. A specific set of Coil 1s was excited as required to generate the non-uniform constant component shown in (b). (c) The reciprocating motion of a single robot was captured as it was propelled along the interface. Scale bar, 1 mm. (d) At different positions (L_r = 20 mm), the robot's midlines within one vibration period were extracted. The symmetry axis of the envelope region was indicated by the black line, with an angle (β_r) against the interface. (e) Relationship between the angle (β_r) and the robot's position (x) during reciprocating motion.

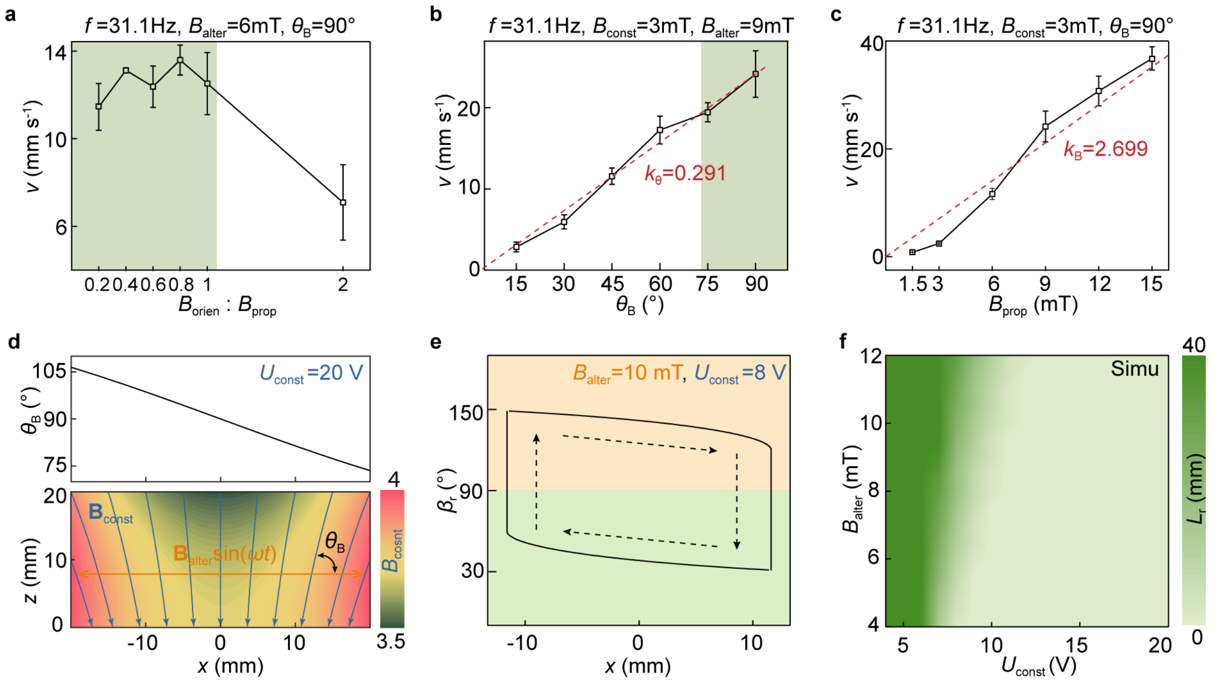

Fig. S25. Model development for the reciprocating motion of robotic agents. The reciprocating motion model of a robot can be established. Experimental data illustrate the relationship between the robot's velocity (v) and magnetic field parameters, including (a) the ratio of flux densities's norm of constant and alternating components ($B_{const} : B_{alter}$), (b) the angle between the constant and alternating components (θ_B), (c) the magnetic flux density of the alternating component (B_{alter}), with a fitted line indicating a proportional increase in speed with θ_B and B_{alter}. Error bars indicate SD. (d) Simulation results depict the distribution of the constant component $\mathbf{B}_{const}$ across the workspace and the angle θ_B between the constant and alternating components. (e) Model-based relationship between the angle (β_r) and the robot's position (x) during reciprocating motion. (f) Model correlating the length of the reciprocating motion region (L_r) with the alternating component flux density (B_{alter}) and the constant excitation voltage (U_{const}).

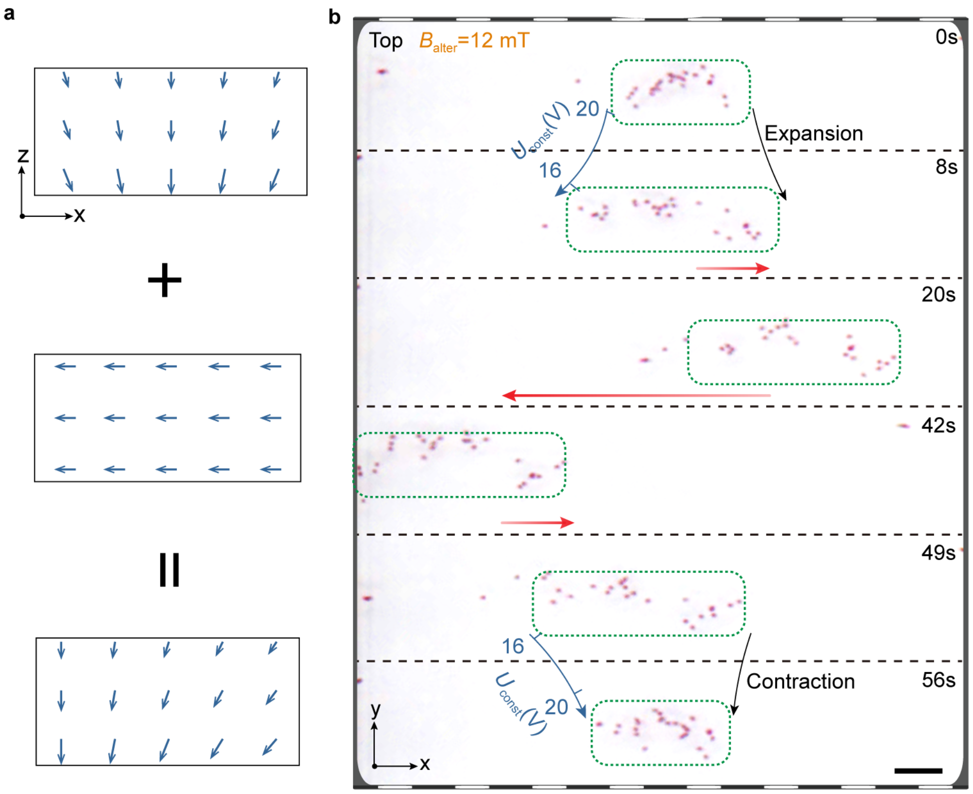

Fig. S26. Combined morphology regulation and locomotion. (a) Shift of the convergence point of the constant magnetic field component. (b)The sequence shows a robot swarm first expanding, then moving, and finally contracting. Scale bars, 5 mm.

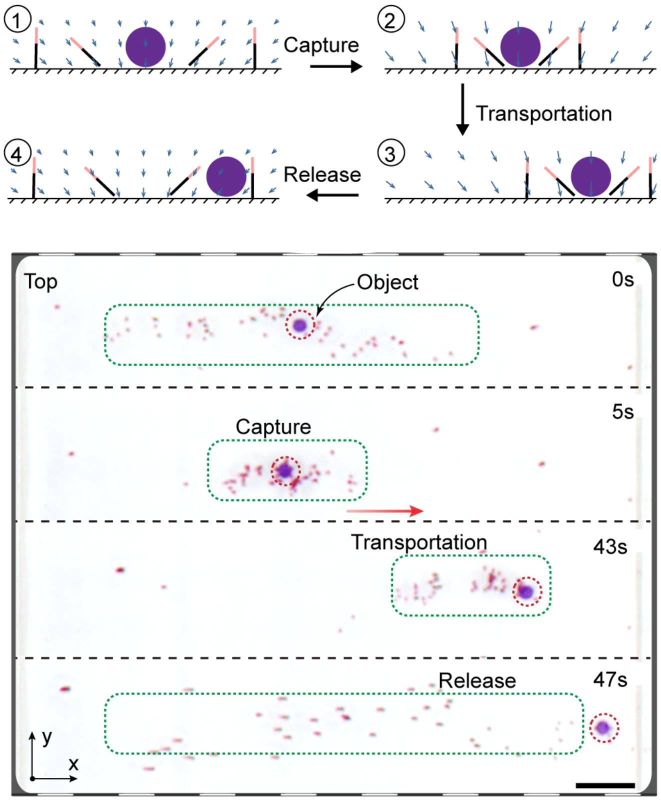

Fig. S27. The robot swarm captures, transports, and then releases a PMMA sphere (5 mm). Scale bar, 10 mm.

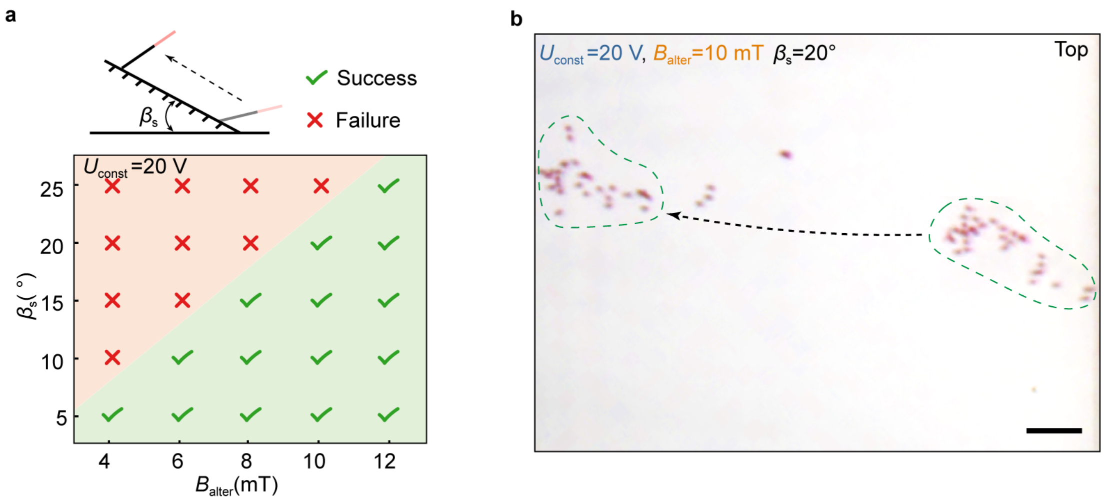

Fig. S28. Adaptability of the robot swarm. (a) Increased adaptability of the robot swarm to steeper inclines by elevating the flux density of the alternating magnetic field component. (b) The robot swarm navigated upward on an inclined surface at a β_s=20° angle. Scale bar, 5 mm.

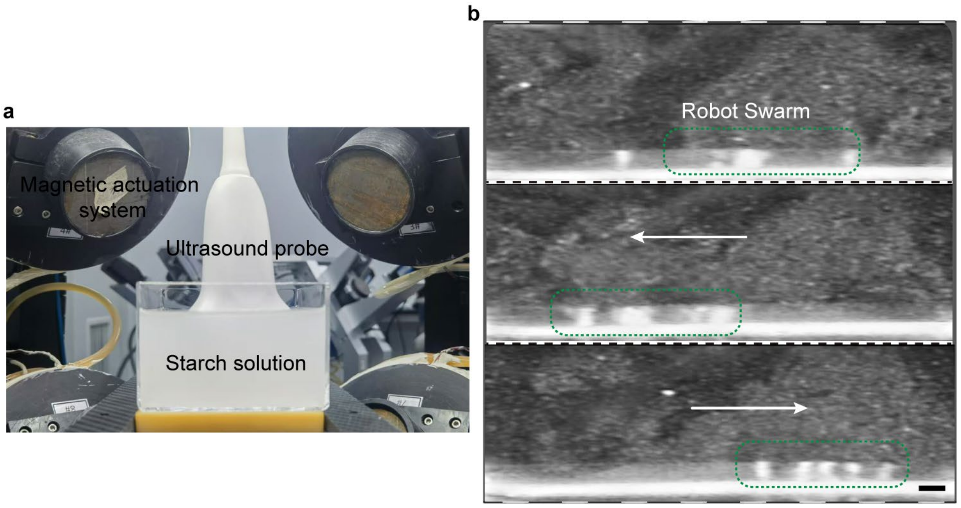

Fig. S29. Ultrasound imaging of the robot swarm's locomotion. (a) Locomotion imaging of a robot swarm in a turbid liquid environment (starch solution) using ultrasound. (b) Controlling the movement of a robot swarm with feedback from ultrasound. Scale bars, 2 mm.

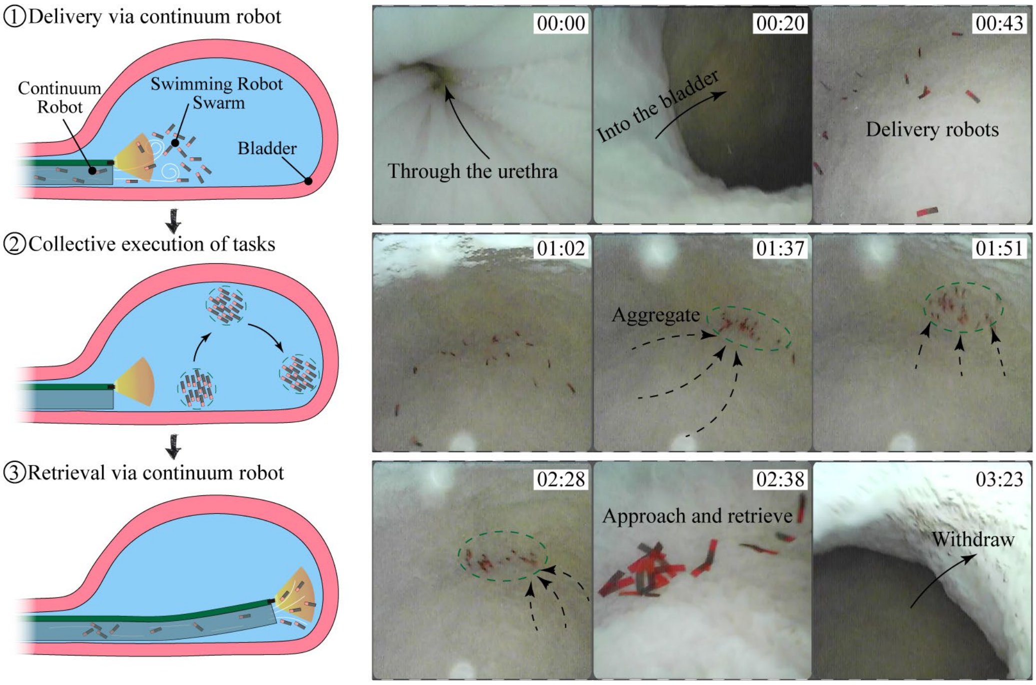

Fig. S30. Delivery of swimming robot warm to the bladder through continuum robots. The ex vivo pig bladder was filled with water as the experimental environment. The continuum robot entered the bladder through the urethra to establish a delivery channel, while the distal camera provided images of the bladder interior. Subsequently, the swimming robot was delivered into the bladder. Under magnetic field control, the swimming robot swarm could freely swim within the bladder, converge toward the target location, and adhere to it. Finally, the swarm converged at a location and was retrieved by the continuum robot.

Supplementary Movies

Movie S1. Release of the swarm of miniature swimming robots. In this demonstrative video, we present the deployment of the “SyncRelease miniature fish-like robot array”, comprising approximately 100 miniature fish-like robots, into an aqueous working environment. Initially bound by a PVA substrate, the robots are liberated as the PVA dissolves within 5 minutes. Under the influence of an externally applied magnetic field, these robots exhibit independent and coordinated motility within the liquid medium.

Movie S2. Close-up and simulation of the individual swimming robot’s motion. We meticulously captured the cyclical motion of the miniature robotic swimmer operating in a magnetic field environment. The oscillating magnetic field induces a rhythmic swinging of the robot’s magnetized head, which, in turn, propels its tail fin to beat against the surrounding fluid for forward propulsion. The simulation results show that the fluid flapped by the tail fin propels the robot forward. The reverse von Karman vortex street wake formed in the tail is a typical thrust-type wake.

Movie S3. Constrained 6-DOF motion of the individual miniature fish-like robot. In Section 1, we demonstrated the proposed robot's ability to rotate (yaw, pitch, and roll) and swim forward through a spiraling 3D trajectory. In Section 2, The robot shows its spatial maneuverability, allowing for horizontal and vertical movements without the need to adjust yaw and pitch angles.

Movie S4. Application of the individual miniature swimming robot with constrained 6-DOF motion. In Section 1, the millimeter swimming robot demonstrates its precision in following predefined trajectories in both planar and three-dimensional spaces. In Sections 2 and 3, we show the environmental adaptation of the individual robot with constrained 6-DOF motion capabilities. The robot can perform vertical translation to overcome gravity when power is insufficient for propulsion. In particular, a robot in upstream swimming can adjust its position while resisting the current through translational motion. Adjusting the position by changing the pitch or yaw angle reduces the forward speed, risking failure in counter-current navigation. In Section 4, we focus on three-dimensional object transport by robots. The robot capable of translational movement can attach an object using its side, capturing it through surface adhesion. Upon reaching the target, an increase in the propulsion field’s amplitude induces larger vibrations in the robot, shaking off the objects for precise delivery. In Section 5, the robot’s capability to adhere to the lesion is demonstrated. It can move horizontally, using its broader sides for better adhesion on lesion surfaces with gradient force.

Movie S5. Controllable 3D aggregation and dispersal of swarm. In Section 1, we illustrate the robot swarm' capability to disperse and aggregate by switching the spatial distribution of the constant component, which is difficult to achieve in a traditional way. In Section 2, the robot swarm performed the sequential aggregation at multiple target locations, guided by the converging magnetic field. In Section 3, The aggregated robotic swarm can disperse and then reaggregate at another target location.

Movie S6. Avoiding obstacles through dispersal and aggregation. In Section 1, the robot swarm dispersed and passed through narrow gaps between multiple parallel obstacles, effectively preventing blockages. In Section 2, the robot swarm aggregated and passed through a passage between two obstacles.

Movie S7. The ex vivo experiment on the aggregation of the robot swarm to lesions. We presented an ex vivo experiment where the robot swarm aggregated toward a gastric lesion and adhered under gradient force.

Movie S8. Morphology regulation, locomotion and manipulation of the robot swarm. In Section 1, we demonstrated the reciprocating motion of the individual robot on a surface. When more robots are introduced, the resulting swarm has the same morphology as the motion area of the individual. In Sections 2, 3, and 4, the morphology and locomotion of the swarm can be regulated by adjusting the magnitude and spatial distribution of the non-uniform constant component. In Section 5, We show the swarm transporting objects on a flat surface.

Movie S9. Formation and locomotion of robot swarms on the space plane. In Section 1, the swarm can move on a sloped surface, which demonstrates its stability and the unaffected control of its morphology and position by the surface's undulations. In Section 2, the swarm can first gather on the ceiling surface and then change position under magnetic control.

Movie S10. Locomotion of the robot swarm in a turbid liquid under ultrasound imaging. We showed the locomotion of a robot swarm in a turbid liquid under ultrasound imaging.

Movie S11. The robot swarm aggregates at the lesion and adjusts to the lesion for better coverage. We demonstrate an ex vivo experiment where the robot swarm aggregated around a lesion site and then adjusted morphology to cover the lesion based on its contours.

Movie S12. Swimming robot swarm performs tasks in the bladder assisted by continuum robots. The ex vivo pig bladder was filled with water as the experimental environment. The continuum robot entered the

bladder through the urethra to establish a delivery channel, while the distal camera provided images of the bladder interior. Subsequently, the swimming robot was delivered into the bladder. Under magnetic field control, the swimming robot swarm could freely swim within the bladder, converge toward the target location, and adhere to it. Finally, the swarm converged at a location and was retrieved by the continuum robot.
